# Self-help mobile messaging intervention for depression among older adults in resource-limited settings: a randomized controlled trial

**DOI:** 10.1038/s41591-024-02864-4

**Published:** 2024-03-14

**Authors:** Marcia Scazufca, Carina Akemi Nakamura, Nadine Seward, Thiago Vinicius Nadaleto Didone, Felipe Azevedo Moretti, Marcelo Oliveira da Costa, Caio Hudson Queiroz de Souza, Gabriel Macias de Oliveira, Monica Souza dos Santos, Luara Aragoni Pereira, Mariana Mendes de Sá Martins, Pepijn van de Ven, William Hollingworth, Tim J. Peters, Ricardo Araya

**Affiliations:** 1 Instituto de Psiquiatria, Hospital das Clinicas HCFMUSP, Faculdade de Medicina, Universidade de São Paulo, São Paulo, Brazil; 2 Departamento de Psiquiatria, Faculdade de Medicina FMUSP, Universidade de São Paulo, São Paulo, Brazil; 3 Health Service and Population Research, Institute of Psychiatry, Psychology and Neuroscience, King’s College London, London, UK; 4 Departamento de Saúde Coletiva, Centro de Ciências da Saúde, Universidade Estadual de Londrina, Londrina, Brazil; 5 Neuroscience Research Group, Institute D’Or for Research and Teaching, São Paulo, Brazil; 6 Health Research Institute, University of Limerick, Limerick, Ireland; 7 Health Economics Bristol, Population Health Sciences, Bristol Medical School, University of Bristol, Bristol, UK; 8 Population Health Sciences, Bristol Medical School, and Bristol Dental School, University of Bristol, Bristol, UK

**Keywords:** Depression, Geriatrics

## Abstract

Scalable solutions to treat depression in older adults in low-resourced settings are urgently needed. The PRODIGITAL-D pragmatic, single-blind, two-arm, individually randomized controlled trial assessed the effectiveness of a mobile messaging psychosocial intervention in improving depressive symptomatology among older adults in socioeconomically deprived areas of Guarulhos, Brazil. Older adults (aged 60+ years) registered with 24 primary care clinics and identified with depressive symptomatology (9-item Patient Health Questionnaire (PHQ-9) scores ≥ 10) received the 6-week Viva Vida intervention based on psychoeducation and behavioral activation (*n* = 298) or a single message (*n* = 305). No health professional support was offered. The primary outcome was improvement from depressive symptomatology (PHQ-9 < 10) at 3 months. Of the 603 participants enrolled (mean age = 65.1 years; 451 (74.8%) women), 527 (87.4%) completed the follow-up assessment. In the intervention arm, 109 of 257 (42.4%) participants had an improved depressive symptomatology, compared with 87 of 270 (32.2%) participants in the control arm (adjusted odds ratio = 1.57; 95% confidence interval = 1.07–2.29; *P* = 0.019). No severe adverse events related to trial participation were observed. These results demonstrate the usefulness of a digital messaging psychosocial intervention in the short-term improvement from depressive symptomatology that can potentially be integrated into primary care programs for treating older adults with depression. Brazilian Registry of Clinical Trials registration: ReBEC (RBR-4c94dtn).

## Abstract (web summary)

The PRODIGITAL-D trial in adults aged 60+ years from socioeconomically deprived areas of Brazil showed that a 6-week self-help mobile messaging psychosocial intervention was effective in improving depression recovery at 3 months compared to a single message control intervention.

## Main

Reducing the burden of depression in older adults is a global health priority, key to ensuring healthy aging and promoting well-being, especially in low- and middle-income countries (LMICs) where 69% of the world’s older population live^1^. In Brazil, the number of adults aged 65 years or older has increased by 57% since 2010, now representing 11% of the total population^2^. Of the older adults interviewed in the Brazilian National Health Survey of 2019, 12% reported that they had experienced depression in the past^3^. Estimates from low-resource settings in Brazil found that 30% of the older population had experienced depressive symptoms suggestive of clinical depression; only one-third of them had received a previous diagnosis^4^.

Programs led by trained nonmental health specialists (task-sharing) and with a team-based approach (collaborative care) have been effective in treating depression in all ages^5,6^, including old age^7–9^. However, these programs often require a prominent role for health professionals, which is not feasible in health systems with few professionals who are already overstretched with other priorities^10,11^. For example, during the coronavirus disease 2019 pandemic, non-coronavirus disease health services were reduced to a minimum in Brazil^12^ because of restrictions on in-person consultations and the health system’s unpreparedness to provide remote care.

A useful strategy to improve access to treatment for depression without increasing health professionals’ workloads is the use of self-help digital mental health interventions^13^, which are low-cost alternatives requiring minimal or no health professional support^14,15^. However, there is little evidence on the effectiveness of such interventions for older adults with depressive symptoms, especially in LMICs^16^.

Therefore, we developed a self-help mobile messaging intervention for older adults with substantial depressive symptoms^17^. Viva Vida is a 6-week digital psychosocial intervention delivered by WhatsApp audio and visual messages, without health professional support. It was adapted from a task-shared, collaborative care psychosocial program led by community health workers in Brazil, PROACTIVE^18^, which improved recovery from depression at the 8-month and 12-month follow-ups^7^. In this study, we evaluated the effectiveness of Viva Vida in a pragmatic, single-blind, two-arm, individually randomized controlled trial among older adults registered in primary care in socioeconomically deprived areas of Guarulhos, Brazil (PRODIGITAL-D)^17^.

## Results

### Participant disposition

**Fig. 1:**
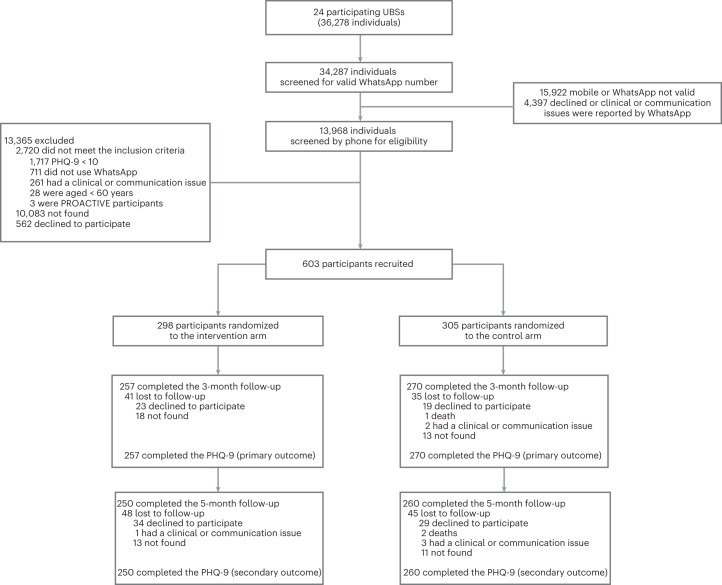
Consolidated Standards of Reporting Trials (CONSORT) diagram.

Participants were enrolled between 8 September 2021 and 8 April 2022. A list with 36,278 eligible older adults registered with the 24 primary care clinics, also called Unidades Básicas de Saúde (UBSs), was provided by the Guarulhos Health Secretariat. Among the 13,968 (38.5%) individuals with a valid WhatsApp number and screened for eligibility by phone: 2,720 (19.5%) did not meet the eligibility criteria (1,717 did not have a depressive symptomatology); 562 (4.0%) declined participation; and 10,083 (72.2%) could not be contacted (they did not answer either of the two calls on different days made by the research assistants) (Fig. 1). A total of 603 participants were recruited, 298 (49.4%) randomized to the intervention arm and 305 (50.6%) to the control arm, with the small discrepancy in numbers attributable to the (blocked) stratified allocation. With variations driven largely by differences in catchment populations, between five and 45 participants were recruited in each UBS, although most of them recruited 20–30 participants.

The descriptive statistics of sociodemographic and other relevant characteristics demonstrated no major differences between trial arms at baseline (Table 1); Extended Data Tables 1 and 2 show that baseline characteristics remained balanced at both follow-up assessments. The 70–79 and 80+ age groups were merged because of the small number of participants aged 80+ years (*n* = 4, all in the control arm). Most participants were aged 60–69 years (83.3%) and women represented 74.8%, of whom most had fewer than 8 years of education (73.8%) and were earning up to a minimum wage (64.3%). The proportion of participants with self-reported hypertension and diabetes were 71.1% and 40.5%, respectively.

**Table 1:** Baseline characteristics in the intervention and control arms

Characteristics	*n* of total sample size (%)	
	Intervention (*n* = 298)	Control (*n* = 305)
Female	225/298 (75.5)	226/305 (74.1)
Age group		
60–69 years	251/298 (84.2)	251/305 (82.3)
70+ years	47/298 (15.8)	54/305 (17.7)
Education		
None	45/297 (15.2)	51/302 (16.9)
1–4 years	83/297 (27.9)	84/302 (27.8)
5–8 years	94/297 (31.6)	85/302 (28.1)
>8 years	75/297 (25.3)	82/302 (27.2)
Personal income^a^		
Up to 1 minimum wage	197/298 (66.1)	191/305 (62.6)
>1–2 minimum wage	60/298 (20.1)	56/305 (18.4)
>2 minimum wage	41/298 (13.8)	58/305 (19.0)
Hypertension (self-reported)	207/298 (69.5)	222/305 (72.8)
Diabetes (self-reported)	123/298 (41.3)	121/305 (39.7)
Receiving pharmacological treatment for depression (self-reported)	38/298 (12.8)	46/305 (15.1)
PHQ-9 severity		
10–14	120/298 (40.3)	119/305 (39.0)
15–19	99/298 (33.2)	100/305 (32.8)
20+	79/298 (26.5)	86/305 (28.2)

^a^In 2021, the minimum wage in Brazil was R$1,100 (approximately US$213).

A total of 527 (87.4%) participants recruited were followed up at 3 months; 41 (13.8%) intervention participants were lost to follow-up, 35 (11.5%) of which were controls. At the 5-month follow-up, 510 (84.6%) of 603 participants were assessed; 48 (16.1%) participants in the intervention arm and 45 (14.8%) participants in the control arm were lost to follow-up. Because the extent of the missing data exceeded the threshold of 10% specified in the statistical analysis plan (SAP), the primary analyses were based on imputed data as presented below alongside (for the primary outcome) the parallel complete case analyses. Extended Data Tables 3 and 4 demonstrate the similarity of baseline demographics between participants with complete data and those with missing data, at the 3-month and 5-month follow-up assessments, respectively.

### Primary outcome

Estimates suggest a beneficial effect of the intervention for the primary outcome of improvement of depressive symptomatology (a 9-item Patient Health Questionnaire (PHQ-9) score < 10) at 3 months. While 109 (42.4%) participants in the intervention arm reached our threshold of improvement, only 87 (32.2%) did so among controls (Table 2); the crude absolute difference was 10.2 (95% confidence interval (CI) = 2.0–18.4) percentage points. The adjusted odds ratio (OR) for this improvement, after imputing missing values, was 1.57 (95% CI = 1.07–2.29; *P* = 0.019). Findings from the complete case analyses were virtually identical (adjusted OR = 1.58, 95% CI = 1.09–2.29; *P* = 0.016); adjusting for the time between randomization and the first follow-up using complete data only also had no appreciable effect on the results (adjusted OR = 1.54, 95% CI = 1.05–2.24; *P* = 0.025).

**Table 2:** Primary (PHQ-9 < 10; improvement) and secondary (PHQ-9 scores reduced by at least 50% between baseline and the first and second follow-up assessments; reduction) outcomes at the 3-month and 5-month follow-ups

Improvement from and reduction in depressive symptomatology at the 3-month and 5-month follow-ups	Intervention	Control			
	*n* of total *n* (%)	*n* of total *n* (%)	Risk difference in percentage points (95% CI)^a^	OR (95% CI)^b,c^	*P*^d^
Primary outcome: improvement from depressive symptomatology at 3 months^e^	109/257 (42.4)	87/270 (32.2)	10.2 (2.0–18.4)	1.57 (1.07–2.29)	0.019
Secondary outcome: improvement from depressive symptomatology at 5 months^e^	127/250 (50.8)	130/260 (50.0)	0.8 (−7.9–9.5)	1.01 (0.70–1.46)	0.938
Secondary outcome: reduction in depressive symptomatology at 3 months^e^	95/257 (37.0)	73/270 (27.0)	9.9 (2.0–17.9)	1.58 (1.08–2.29)	0.016
Secondary outcome: reduction in depressive symptomatology at 5 months^e^	111/250 (44.4)	112/260 (43.1)	1.3 (−7.3–9.9)	1.04 (0.73–1.48)	0.841

^a^These (post hoc) analyses are based on crude (that is, unadjusted) data on a complete case basis.

^b^ORs and 95% CIs were calculated using fixed-effects logistic regression models, from which the *P* values were derived.

^c^All estimates had missing data imputed according to trial arm using the multiple imputation by chained equations (MICE) models, which included stratification (sex, age group and baseline severity of PHQ-9 score (categorical)) and predictors of missingness (extended data).

^d^All *P* values reported are based on two-sided Wald tests from the relevant logistic regression model, with no adjustments for multiple tests.

^e^The primary and secondary outcomes of improvement from depressive symptomatology were defined as PHQ-9 scores less than 10; the secondary outcome of reduction in depressive symptomatology was defined as PHQ-9 scores reducing by at least 50% between baseline and the first and second follow-up assessments (yes/no).

### Secondary outcomes

At 5 months, virtually identical proportions had improved in the two groups (Table 2); 50.8% (*n* = 127) and 50.0% (*n* = 130) for the intervention and control arms, respectively (adjusted OR = 1.01; 95% CI = 0.70–1.46; *P* = 0.938). Table 2 shows that the secondary outcome of PHQ-9 scores reducing by at least 50% from baseline values also demonstrated a benefit of the intervention at 3 months (1.58; 95% CI = 1.08–2.29; *P* = 0.016), but not at 5 months (1.04; 95% CI = 0.73–1.48; *P* = 0.841). The same general conclusions can be drawn from the (post hoc) analyses of the effects presented in terms of (crude) absolute risk differences (Table 2). Complete case analyses again showed extremely similar results (Extended Data Table 5). There was no evidence to support an effect of the intervention for any other secondary outcome at the 3-month and 5-month follow-up assessments using the imputed data: PHQ-9 scores (−1.14, 95% CI = −2.33–0.05; *P* = 0.060 and −0.21, 95% CI = −1.36–0.94; *P* = 0.716), 7-item General Anxiety Disorder (GAD-7) questionnaire scores (−0.89, 95% CI = −1.88–0.11; *P* = 0.082 and −0.14, 95% CI = −1.20–0.92; *P* = 0.793), 5-level European Quality of Life five-dimensional questionnaire (EQ-5D-5L) scores (0.025, 95% CI = −0.015–0.064; *P* = 0.218 and 0.035, 95% CI = −0.001– 0.071; *P* = 0.059), ICEpop CAPability measure for older people (ICECAP-O) scores (0.018, 95% CI = −0.009–0.045; *P* = 0.185 and 0.002, 95% CI = −0.024–0.029; *P* = 0.856), 3-item University of California, Los Angeles (UCLA) scores (0.19, 95% CI = −0.09–0.48; *P* = 0.186 and 0.22, 95% CI = −0.07–0.52; *P* = 0.140) (Table 3). Similar results were found using the complete cases (Extended Data Table 6).

**Table 3:** Secondary outcomes, including depressive symptomatology (PHQ-9), anxiety symptomatology (GAD-7), health-related quality of life (EQ-5D-5L), capability well-being in older adults (ICECAP-O) and perceived loneliness (3-item UCLA) at the 3-month and 5-month follow-ups

Outcome	Baseline		3-month follow-up				5-month follow-up			
	Intervention	Control	Intervention	Control	Coefficient (95% CI)^a,b^	*P*^c^	Intervention	Control	Coefficient (95% CI)^a,b^	*P*^c^
PHQ-9, *n*	298	305	257	270			250	260		
Mean (s.d.)	16.39 (4.66)	16.68 (4.83)	11.72 (7.72)	12.92 (7.13)	−1.14 (−2.33–0.05)	0.060	10.01 (7.07)	10.41 (7.01)	−0.21 (−1.36–0.94)	0.716
GAD-7, *n*	285	283	256	268			250	260		
Mean (s.d.)	14.46 (4.62)	14.36 (4.75)	10.30 (6.62)	11.07 (6.43)	−0.89 (−1.88–0.11)	0.082	9.40 (6.79)	9.49 (6.66)	−0.14 (−1.20–0.92)	0.793
EQ-5D-5L, *n*	296	305	256	266			247	259		
Mean (s.d.)	0.789 (0.182)	0.794 (0.170)	0.812 (0.189)	0.801 (0.171)	0.025 (−0.015–0.064)	0.218	0.840 (0.164)	0.825 (0.171)	0.035 (−0.001–0.071)	0.059
ICECAP-O, *n*	295	300	254	266			246	259		
Mean (s.d.)	0.614 (0.172)	0.621 (0.190)	0.655 (0.174)	0.642 (0.194)	0.018 (−0.009–0.045)	0.185	0.665 (0.165)	0.667 (0.172)	0.002 (−0.024–0.029)	0.856
3-item UCLA, *n*	298	305	255	267			250	259		
Mean (s.d.)	6.04 (1.91)	6.16 (1.92)	5.68 (1.97)	5.54 (1.94)	0.19 (−0.09–0.48)	0.186	5.43 (1.97)	5.25 (1.97)	0.22 (−0.07–0.52)	0.140

PHQ-9 scores range from 0 to 27, with higher scores representing more severe depressive symptomatology.

GAD-7 scores range from 0 to 21, with higher scores representing more severe anxiety symptomatology.

EQ-5D-5L scores range from −0.264 to 1, with higher scores representing higher quality of life.

ICECAP-O scores range from 0 to 1, with higher scores representing greater levels of capability well-being.

The 3-item UCLA scores range from 3 to 9, with higher scores representing more severe levels of loneliness.

^a^Differences in means were estimated using linear regression models, adjusted for the baseline assessment of the corresponding outcome and the stratified variable (sex, age group and baseline severity of the PHQ-9 score (categorical))

^b^All estimates had missing data imputed separately, according to trial arm, using MICE models that included predictors of missingness (extended data), stratification (sex, age group and baseline severity of PHQ-9 score (categorical)) and any imbalances in outcome measure at baseline.

^c^All *P* values reported are based on two-sided *t*-tests from the relevant linear regression model, with no adjustments for multiple tests.

### Safety

Follow-up data showed that six participants (2.0%) in each arm reported suicidal ideation. The safety protocol described in Methods was applied. Eighteen intervention participants (6.0%) and 20 controls (6.6%) were hospitalized during the trial for reasons not related to mental health. Two deaths (0.7%) were reported in the control arm. All events were considered not related to either the intervention or study participation.

### Exploratory analyses

Bearing in mind the number of such tests performed, there was no evidence of differential effects in the prespecified subgroup analyses, with *P* values ranging from 0.055 to 0.953 (Extended Data Table 7).

The electronic system indicated that 226 (75.8%) intervention participants ‘opened’ at least 36 of the 48 messages (our prespecified minimum therapeutic dose), 237 (79.5%) opened at least 24 messages and 173 (58.1%) opened all 48 messages.

The main complier average causal effect (CACE) analysis did not yield strong evidence of an improvement in mean PHQ-9 scores for participants who opened 36+ of the messages compared with others (Table 4) at either the 3-month or 5-month follow-ups. Nonetheless, the *P* values for the main and sensitivity CACE analyses at 3 months (*P* = 0.054 for the prespecified threshold) provide marginal evidence that the adjusted effect of the intervention increased as the proportion of opened messages increased (Table 4).

**Table 4:** CACE analyses on the mean PHQ-9 scores at the 3-month and 5-month follow-ups using electronic recordings

Threshold	3-month follow-up		5-month follow-up	
	Adjusted difference in mean PHQ-9 (95% CI)^a,b^	*P*^c^	Adjusted difference in mean PHQ-9 (95% CI)^a,b^	*P*^c^
Opened at least 24 messages (*n* = 237, 79.5%)	−1.52 (−3.06–0.02)	0.053	−0.49 (−1.98–0.99)	0.513
Opened at least 36 messages, that is, the minimum therapeutic dose (*n* = 226, 75.8%)	−1.59 (−3.21–0.02)	0.054	−0.52 (−2.08–1.04)	0.513
Opened all 48 messages (*n* = 173, 58.1%)	−2.09 (−4.20–0.03)	0.053	−0.68 (−2.71–1.35)	0.513

PHQ-9 scores range from 0 to 27, with higher scores representing more severe depressive symptomatology.

^a^All models are adjusted for stratification (sex, age group and baseline severity of PHQ-9 score (categorical)).

^b^All estimates had missing data imputed separately, according to trial arm, using MICE models that included predictors of missingness (extended data), stratification (sex, age group and baseline severity of PHQ-9 score (categorical)).

^c^All *P* values reported are based on two-sided *t*-tests from the relevant CACE regression model, with no adjustments for multiple tests.

### Sensitivity analyses

The sensitivity analyses relating to aspects, such as the timing of the follow-up and altering the thresholds for the CACE analyses, have been covered in the relevant sections above. Results from the sensitivity analysis testing the missing at random (MAR) assumption for the primary and secondary outcomes of improvement from depressive symptomatology at 3 and 5 months suggest that estimates moved toward the null hypothesis. In other words, participants who improved from depressive symptomatology were more likely to be missing at the follow-up, compared with participants who did not improve (Extended Data Table 8).

## Discussion

This RCT assessed a self-help digital psychosocial intervention delivered by WhatsApp to older adults in socioeconomically deprived areas in Brazil. Compared with a single educational message, at the primary (3-month) follow-up, the 6-week Viva Vida program showed increased odds of improvement from depressive symptomatology and greater likelihood of a reduction in PHQ-9 of at least 50%. No such differences were observed at 5 months. Levels of anxiety symptomatology, loneliness, quality of life and capability well-being were similar in the two arms at both follow-ups. Equally important, this study demonstrated the feasibility and acceptability of a simple and affordable intervention that can provide help to a large proportion of older adults with depressive symptoms in Brazil. This would be a major achievement in a country with a large treatment gap and lack of specialists to cover such a need.

Digital strategies to deliver behavioral activation interventions have achieved small-to-medium effects on depression, anxiety and quality of life in adults^19^. Similar to our findings, two trials (CONEMO) conducted in adults with depression and comorbid hypertension or diabetes in Brazil and Peru showed that a 6-week digital intervention minimally supported by nurses or nurse assistants improved depressive symptomatology by at least 50% at 3, but not 6 months, compared with enhanced usual care^20^. The studies suggest that both the CONEMO and Viva Vida digital interventions, based on behavioral activation, accelerate the improvement from depression in the short-term in middle-to-older adults but the clinical differences with control groups decrease mainly because these comparison groups show improved outcomes through time. Further studies could evaluate if booster messages in the active group (Viva Vida) would help maintain the speed of improvement and increase clinical benefits even further over time, so that the earlier differences with the control remain over a longer time.

Human support, such nurse assistants in CONEMO, has been reported as an important component of effective digital mental health interventions^16^. However, the Viva Vida program precluded such clinical support because a self-help mental health intervention could provide a more affordable first step for treating depression in primary care. According to the updated taxonomy of digital mental health interventions by Pineda et al.^13^, the Viva Vida program is classified as a type 3 intervention because it offered technical support during the trial along with other trial procedures to guarantee the delivery and fidelity of the intervention, and the reliability of the data collected. Nevertheless, it can be turned into a type 4 fully automated intervention in the real world, where such controlled procedures are not necessary. The messages can be initially accessed from anywhere with an internet connection; once the content is downloaded to WhatsApp or to a device, the messages can be accessed at any time even without an internet connection. With such a fully automated system in place, a more affordable program can be delivered to a large number of people with a small incremental cost for each message sent.

The estimated intervention effect (10.2 percentage points in absolute terms) was lower than that specified as the original target difference (15 percentage points). As the target difference was based on collaborative care interventions with the active support of primary care professionals, we contend that our findings are important because a digital intervention would reach many of those who at the moment are receiving no help. In low-resource settings, where access to mental health care is very limited, fully automated programs might be used to cover treatment gaps when there are limited or no other alternatives. We do not expect it to be suitable for everyone and some individuals might need to be referred to a more intense program with professional support. Moreover, while from the various confidence intervals we cannot rule out smaller, arguably unimportant effects (relative or absolute), it is likewise the case that absolute effects at least as large as our target difference are contained in the relevant interval estimates at 3 months. The effect size of our secondary depression outcomes at 3 months are nonetheless similar to those for digital interventions supported by nurse assistants^20^ or lay counselors^21^.

WhatsApp is widely used by the target population in Brazil. The use of a well-known and accepted messaging application enables the receipt of messages without requiring the download of a new application or the acquisition of additional digital skills, and is consistent with the findings of a systematic review of similar technologies^16^. Older adults with relatively low digital literacy level or without access to a high-speed internet connection were able to use this tool, which showed high adherence and acceptability. Women represented 75% of our sample as a reflection of the epidemiological profile of depressive symptoms among the older population^4,22^. Future studies could explore the effects of personalized programs according to different participants’ preferences or characteristics, such as sex and presence of other comorbidities.

In addition to the issue of the lower magnitude of the observed difference in absolute terms (and some of the values in the relevant CI) compared with the target difference as discussed above, this study has some important limitations. As assessments were done by phone, simple questions and short interviews were the best option available. Thus, the use of the PHQ-9 for the assessment of depressive symptomatology was a pragmatic choice and the threshold of ten as indicative of clinical depression was based on the literature^23,24^. Relevant conditions among older adults, such as diabetes and hypertension, were self-reported. We were unable to assess cognition with a standardized assessment over the phone. However, research assistants were trained to identify when the older adult had substantial difficulties understanding and answering the questions. In these situations, the assessment was interrupted. When possible, the research assistant asked permission to speak to someone in the household about the difficulties presented by the person during the interview. In addition, the research assistant discussed these cases with the study coordinator, who decided whether the person would be included in the study. During the interview, we asked if the person had any vision or hearing problems, and if they had any difficulty in understanding audio or visual messages on a mobile phone. Individuals with a lower digital literacy or without access to WhatsApp were not included in this study because of the design of the digital intervention. Major adaptations are needed to deliver a more appropriate intervention to reach such individuals. Finally, cost-effectiveness and process evaluation remain to be assessed using the economic and qualitative data collected within PRODIGITAL-D. Indeed, the qualitative data may also shed further light on the issue of any effects of opening more rather than fewer of the messages as investigated within the above, essentially exploratory, CACE analyses.

Albeit at about half the level that we accounted for in our sample size calculation for the primary outcome at 3 months (about 12.5% overall compared with the 25% allowed for), a further limitation is that there is a risk of bias through attrition. As well as being low compared with expectations, some reassurance on this issue can be gleaned from the similar amounts of missing data across the two arms and the consistency of results between the analyses using imputed data (albeit under an MAR assumption) and the complete case analyses.

As this was a research project, we used standardized procedures for assessments, including quality control measures. Trained research assistants performed the recruitment and outcome assessments remotely on participants of both study arms. It is always possible that simply asking questions may have a therapeutic effect. In any case, the same procedures were used with participants in both arms. Another procedure common to participants of both arms was the contact from the technical support team if they experienced difficulties accessing messages. Such contacts were not frequent (20 in the intervention arm and 12 in the control arm) and were strictly for solving any technical problems with receiving messages. Participants in the intervention arm could also contact the technical support team during the 6-week intervention, but no contacts were made. In any case, it was not possible to disentangle the effect of the intervention from the possible therapeutic effects that remote assessments or the technical support contact may have had on participants.

This RCT provides evidence of the feasibility and a small and short-term effectiveness of a simple, brief and well-accepted self-help digital intervention for depressive symptomatology in older adults that does not require the active support of health professionals. Such interventions, which can be accessed from anywhere with (and without) an internet connection, can contribute to mental health care in low-resourced settings where there is little access to treatment for depression.

## Methods

### Study design and participants

PRODIGITAL-D was a pragmatic, single-blind, two-arm individually RCT with 1:1 allocation, conducted in 24 primary care clinics, also called UBSs, in underprivileged areas of the city of Guarulhos, Brazil. This city is part of the metropolitan region of São Paulo with a population of around 1.3 million.

We recruited individuals aged 60+ years, registered with any of the participating 24 UBSs, able to receive WhatsApp messages, and who screened positive for depressive symptomatology (PHQ-9 ≥ 10)^23^ with at least one core symptom of depression (low mood or anhedonia, that is, PHQ-2 ≥ 1)^25^. The PHQ-9 threshold had a sensitivity of 91% and specificity of 88% against diagnostic interviews for adults aged 60+ years^24^. Exclusion criteria included substantial visual or hearing impairments that would hinder comprehension of messages on a mobile phone, acute suicidal risk identified during the screening assessment, another individual residing in the same household already participating in PRODIGITAL-D or previous participation in the PROACTIVE trial. Informed verbal consent was obtained before the screening assessment and when participants were invited to the trial, both conducted by phone. Consent was audio-recorded after authorization from the older adult. Details of the protocol are published elsewhere^17^.

This study was approved by the ethics committee of the Hospital das Clínicas da Faculdade de Medicina da Universidade de São Paulo (Comissão para Análise de Projetos de Pesquisa, ref: 4.097.596, first approved 10 March 2021) and authorized by the Guarulhos Health Secretary. It was registered with the Brazilian Registry of Clinical Trials (ReBEC), RBR-4c94dtn, on 22 October 2021. Trial registration materials were submitted to ReBEC on 3 August 2021.

### Procedures

A list with all the names and phone numbers of older adults registered with the 24 participating UBSs was provided by the Guarulhos Health Secretariat. After excluding duplicates and individuals without phone numbers, individuals were assigned a random ID for our study, as the original list was in alphabetical order. Research assistants then sent a WhatsApp message to older adults according to the new list sorted by ascending random ID numbers to search for individuals with a valid mobile number and who used WhatsApp. Individuals with a valid WhatsApp number (that is, who received the prescreening WhatsApp message) were approached by telephone to be screened further for depressive symptomatology (using the PHQ-2)^25^. Up to two call attempts were made on different days. Individuals with a PHQ-2 score of one or greater were then asked the other seven items of the PHQ-9 questionnaire^23^. Those with PHQ-9 scores of ten or greater completed a baseline assessment to ascertain the exclusion criteria; eligible individuals were invited to participate in the trial. The screening and baseline assessments together lasted approximately 35 min. The invitation for the trial, which includes reading the full participant information sheet, occurred in the same call, whenever the individual agreed to continue it for around 15 min longer. When this was not possible, an additional call no more than 28 days after the PHQ-9 screening was made to complete the recruitment. Follow-up data were collected by phone at 3 (weeks 12–16) and 5 months (weeks 20–24) after sending the first message (intervention) or the single message (control). A 4-week window was used to maximize response to follow-up considering the logistical realities of conducting phone interviews.

Quality control of a random sample of recorded assessments was conducted by an independent research assistant (not involved in any data collection) to ensure all procedures had been followed and the quality of data collected.

### Randomization and masking

Participants were stratified according to age group (60–69 years, 70–79 years, 80+ years), sex (male, female) and severity of depressive symptoms (baseline PHQ-9 categories: 10–14, 15–19, 20+). The allocation sequence was generated using randomly permuted blocks with random block sizes of six, eight or ten by research team members not involved in data collection (C.A.N. and T.J.P.). The ‘randomization module’ of the Research Electronic Data Capture (REDCap)^26,27^ was used to conceal the allocation sequence and randomize individuals.

Research assistants involved in recruitment and follow-up data collection were blinded to trial allocation. Two different groups of research assistants were responsible for either the first or the second follow-up assessment. Researchers responsible for scheduling the automated delivery of messages were not blinded to trial allocation but had no role in collecting the inclusion or outcome data. Due to the nature of the interventions, it was not possible to mask trial participants.

### Interventions

Participants in both arms continued receiving their UBS’s usual primary care. The research team did not interfere with that care (consultation or treatments, including medication) and the UBS health professionals had no role in Viva Vida.

During the first 2 weeks (for the intervention arm) and after the single message (for the control arm), a technical support person identified and contacted individuals who were having issues with receiving or opening the message. This was to help participants with any potential technical issues that were preventing them from accessing the messages.

### Viva Vida program

**Extended Data Fig. 1:**
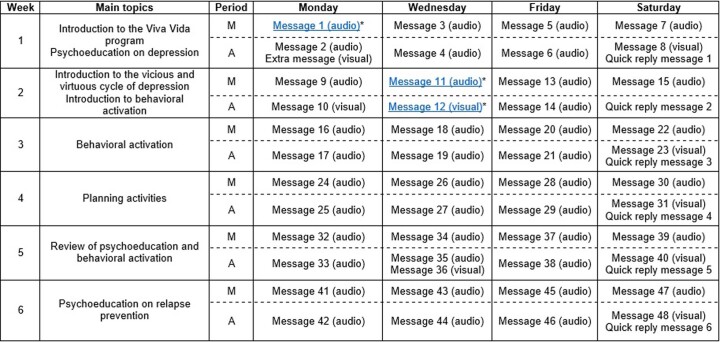
Structure of the Viva Vida program. *Abbreviations*: A: afternoon; M: morning. * Example messages with corresponding links are provided for reference - Message 1 (audio), Message 11 (audio) and Message 12 (visual). Week 1: Introduction to the Viva Viva Vida program. Psychoeducation about depression. Extra message with contact of technical support. Week 2: Introduction to the vicious and virtuous cycle of depression, using the metaphor of the ‘Wheel of improvement and worsening’. Introduction to behavioral activation using examples of simple things that can help to feel better about the common symptoms of depression. Week 3: Continues behavioral activation using examples of simple things that can help you feel better about the common symptoms of depression. Week 4: Planning activities, the importance of choosing pleasant and meaningful activities, and how to organise the activities during the week. Week 5: Review of psychoeducation and behavioural activation contents. Week 6: Psychoeducation on relapse prevention, keeping in the virtuous cycle of depression after the end of the program and the importance of early identification of recurrence of depressive symptoms.

Participants allocated to the intervention received a psychosocial intervention delivered via WhatsApp. A total of 48 audio or visual messages were automatically sent 4 days a week for 6 weeks (one in the morning, one in the afternoon). The audio messages were based on psychoeducation^28^ or behavioral activation^29^ principles and lasted on average 3 min using storytelling techniques. In the audio messages, there were two characters (Ms Ana and Mr Leo) reading and commenting on letters sent by fictional older adults participating in the program. Through these letters they shared stories about their lives and symptoms of depression and how the Viva Vida intervention helped them to feel better. Eight visual messages including summary diagrams were used to reinforce the content of the audio messages. Additionally, at the end of each week, participants received an extra message with a question about their opinion of the program that could be responded to using the WhatsApp quick reply tool. Participants also received one message at the beginning of Viva Vida with a support contact number in case they experienced technical issues receiving the messages. As the messages were delivered automatically, this strategy allowed participants to contact us in case they were not receiving the intervention as intended. During the program, participants were also advised to visit the UBS if they needed professional support for any health issues. Extended Data Fig. 1 shows the structure of the Viva Vida program and the content of the audio and visual messages that participants received each week.

The Viva Vida messages were based on the PROACTIVE intervention delivered to older adults^10,18,30^. The use of WhatsApp as a tool to deliver an intervention was initially discussed in a group with six older adults and one community health worker in one UBS. After developing the messages, selected older adults received a sample and were interviewed to evaluate qualitatively the acceptability of the messages. The automated system and the recruitment by phone were subsequently evaluated in a feasibility study with a random sample of older adults with depressive symptomatology recruited from two UBSs in Guarulhos that did not participate in the study.

### Single message control arm

Control arm participants were sent a single and brief (6-min) audio message providing information about depression and simple ways to deal with depressive symptoms. It also offered additional tips encouraging participants to live a healthier life, such as improving sleep and maintaining a balanced diet, advising them to seek health care if they felt they needed additional support.

### Outcomes

Improvement from depressive symptomatology at 3 months was the primary outcome. We considered improvement when the PHQ-9 scores were below 10 (ref. ^23^). Secondary outcomes included improvement from depressive symptomatology at 5 months and reduction in PHQ-9 scores by at least 50% between baseline and follow-up visits at 3 and 5 months. We also evaluated as exploratory outcomes the effects on the continuous scores of depressive symptomatology (PHQ-9), anxiety symptomatology with the GAD-7 (ref. ^31^), loneliness with the 3-item UCLA loneliness scale^32^, health-related quality of life EQ-5D-5L^33^ and capability well-being with the ICECAP-O^34^.

### Safety

As mentioned before, participants continued receiving usual primary care and were advised to contact the primary care team whenever they felt they needed additional support from health professionals. The risk associated with receiving the interventions was considered minimal. Severe adverse events, including acute suicidal risk, hospitalizations and death were collected during the follow-up assessments using a standardized form. Participants who reported suicidal ideation were assessed for suicidal risk with the standardized protocol used in a previous study^7^. UBS managers were contacted to inform about participants with suicidal ideation. We also investigated any hospitalization or death with participants or family members to understand if it was related to study participation.

### Data collection and management

All the measures mentioned above were applied at baseline, and at the 3-month and 5-month follow-up assessments. Sociodemographic and clinical characteristics were also collected at baseline. Economic data were collected at each follow-up assessment. Severe adverse events were assessed for all participants at 3 and 5 months.

Data were collected and managed using REDCap^26,27^ hosted at the Hospital das Clínicas da Faculdade de Medicina da Universidade de São Paulo. REDCap is a secure, web-based software platform designed to support data capture for research studies. A system was developed to collect and store data from the WhatsApp messages. A dedicated server component scheduled the messages for groups of participants (grouped according to trial arm and the start date of the messages based on the date of randomization) and pushed these to the participants using the WhatsApp Business interface. The latter provides time stamps indicating when participants receive and open their messages, as well as the answers chosen using the quick reply tool and the content of spontaneous messages sent by participants.

### Sample size

With an assumed attrition of 25%, 440–500 randomized individuals would yield 80–85% power to detect a 15 percentage point difference in depression improvement (PHQ-9 < 10) rates between the control and intervention arms at 3 months (25% versus 40%) using a two-sided 5% alpha. Such a difference in improvement rate is considered clinically meaningful^8,30^ but there were no local studies that had investigated a clinically meaningful threshold.

### Statistical analysis

Descriptive statistics were obtained using complete cases. All comparative analyses were performed using intention-to-treat principles with imputed data in regression models, aligned to the SAP contingent on the amount of missing data^35^. Logistic regression was used for binary outcomes; linear regression was used for continuous outcomes. All regression analyses were adjusted for stratification, and all analyses with an outcome other than PHQ-9 were also adjusted for the baseline score of the corresponding outcome.

Prespecified subgroup analyses at both follow-up assessments were investigated using likelihood ratio tests for interactions between randomized arm and the following: sex; age; educational level; comorbid physical illness (hypertension, diabetes or both); and baseline PHQ-9 severity categories. We also ran a model for the primary outcome adjusting for elapsed time between consenting to participate in the trial and the first or primary follow-up.

Initially, patterns of missingness were investigated by comparing missing with complete data for the baseline demographic characteristics, according to trial arm, at both follow-up visits. To reduce bias and loss of information, we used MICE with 50 imputations, as implemented in the multiple imputation command in Stata under the assumption that data were MAR^36^. Variables included in the MICE models consist of the ‘improved from depression’ outcome, covariates described in the SAP (age group, severity of PHQ-9, sex, treatment arm) and variables found to be predictors of missingness^37,38^. Predictors of missingness included the following: hypertension at baseline, income and levels of enjoyable activities participants engaged in. Also included were baseline scores for corresponding outcome models, including depressive symptomatology (PHQ-9), anxiety symptomatology (GAD-7), health-related quality of life (EQ-5D-5L), capability well-being (ICECAP-O) and loneliness (3-item UCLA).

To assess the sensitivity of our findings against modest departures from the MAR assumption, a weighted sensitivity analysis using the selection model approach was applied^39–41^. Briefly, once data had been imputed under MAR, parameter estimates from each imputed dataset were reweighted to allow for the data to be missing not at random. To test the stability of our model, we considered different degrees of departure from the MAR assumption by considering plausible values of *δ* ranging from 0.10 to 0.40. This range corresponds to ORs for the data being observed when a participant improved from depression compared to when they did not, ranging from 1.11 to 1.50 (that is, the exponential of 0.10 and 0.40 respectively).

CACE analysis^42^ using an instrumental variable estimator and imputed data was applied to estimate the effect of the number of messages electronically recorded as being opened on depression outcomes. The protocol^17^ and the SAP^35^ prespecified the ‘minimum therapeutic dose’ as listening to ‘most of the messages’ and ‘more than half of the messages’ received, respectively because it was initially based on self-reported categories (‘none’, ‘a few’, ‘at least half’, ‘most of the messages’ and ‘all of the messages’) of a question from the first follow-up assessment. Because in the event we were able to access the electronic data, we operationalized the prespecified minimum therapeutic dose as opening 36 or more messages (75% of the total of 48 messages) versus opening 35 or fewer messages. The CACE analyses were conducted using the PHQ-9 score at 3 and 5 months, adjusting for stratification. We also conducted sensitivity analyses using thresholds of opening at least half of the total messages (24+) versus 23 messages or fewer, and opening all 48 messages versus not doing so.

Regression diagnostics were run for all regression models. The Box–Tidwell test was run after the logistic regression models to test whether the logit transform was a linear function of the predictors for the different models. Normality assumptions for linear regression models were evaluated through residual plots. Statistical tests were two-sided and all analyses were conducted using Stata v.17 (StataCorp LLC).

### Ethics and inclusion statement

Most of the authors (10 of 15) are from Brazil, where the RCT took place, and were funded to work on the development and delivery of the intervention, data collection or study management. The authors based in high-income countries are researchers who contributed to the conception of the study and supported the application to obtain funding shared between Brazil and the UK. This study is relevant for Brazil and other LMICs. One member of the trial steering committee is from the Guarulhos Health Secretary’s office, while two other members are researchers based in other cities of Brazil. Meetings with primary care and UBS managers in Guarulhos were held before, during and after the trial. This study was approved by the ethics committee of the Hospital das Clínicas da Faculdade de Medicina da Universidade de São Paulo (Comissão para Análise de Projetos de Pesquisa, ref. 4.097.596) and authorized by the Guarulhos Health Secretary.

### Reporting summary

Further information on research design is available in the Nature Portfolio Reporting Summary linked to this article.

## Online content

Any methods, additional references, Nature Portfolio reporting summaries, source data, extended data, supplementary information, acknowledgements, peer review information; details of author contributions and competing interests; and statements of data and code availability are available at 10.1038/s41591-024-02864-4.

### Supplementary information

Supplementary Information Trial protocol.

Reporting Summary

## Figures and Tables

**Extended Data Table 1:**
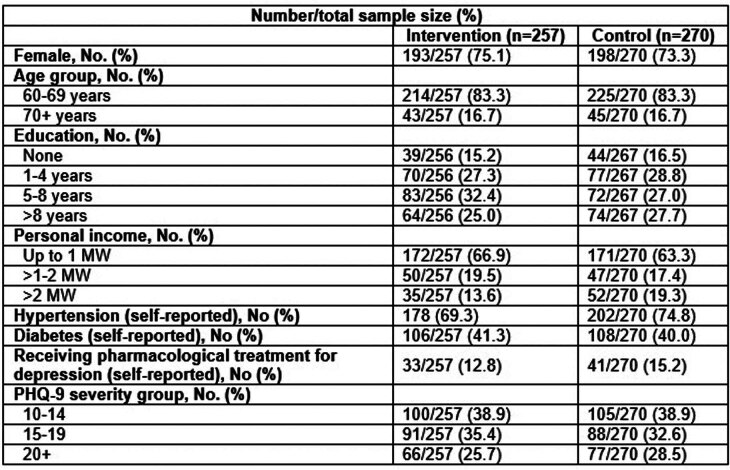
Comparison of baseline demographics between trial arms for participants followed up at 3 months

MW, minimum wage (in 2021, the minimum wage in Brazil was R$1,100 (approximately US$213)); PHQ-9, 9-item Patient Health Questionnaire.

**Extended Data Table 2:**
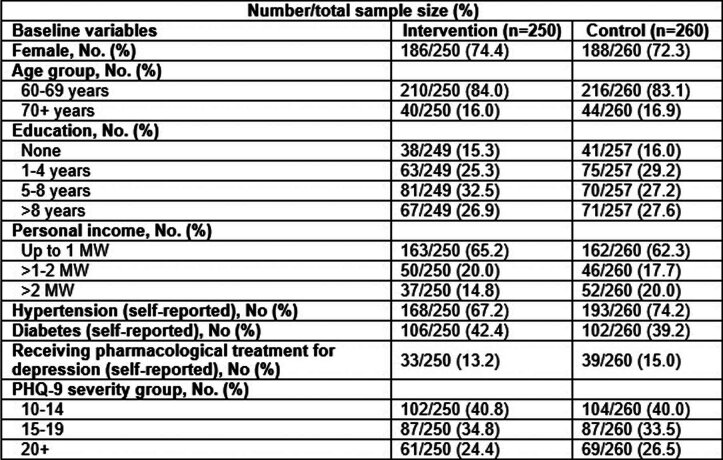
Comparison of baseline demographics between trial arms for participants followed up at 5 months

MW, minimum wage (in 2021, the minimum wage in Brazil was R$1,100 (approximately US$213)); PHQ-9, 9-item Patient Health Questionnaire.

**Extended Data Table 3:**
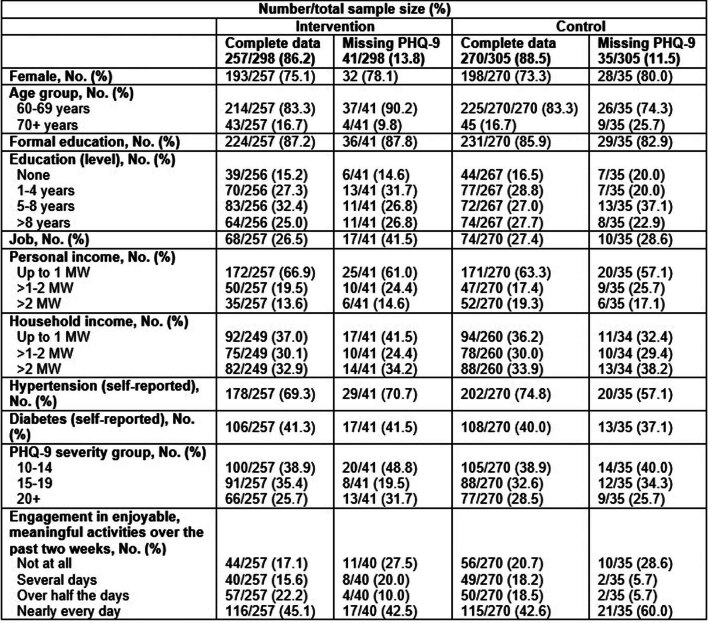
Comparison between participants with complete data and participants with missing data at the 3-month follow-up assessment

MW, minimum wage (in 2021, the minimum wage in Brazil was R$1,100 (approximately US$213)); PHQ-9, 9-item Patient Health Questionnaire.

**Extended Data Table 4:**
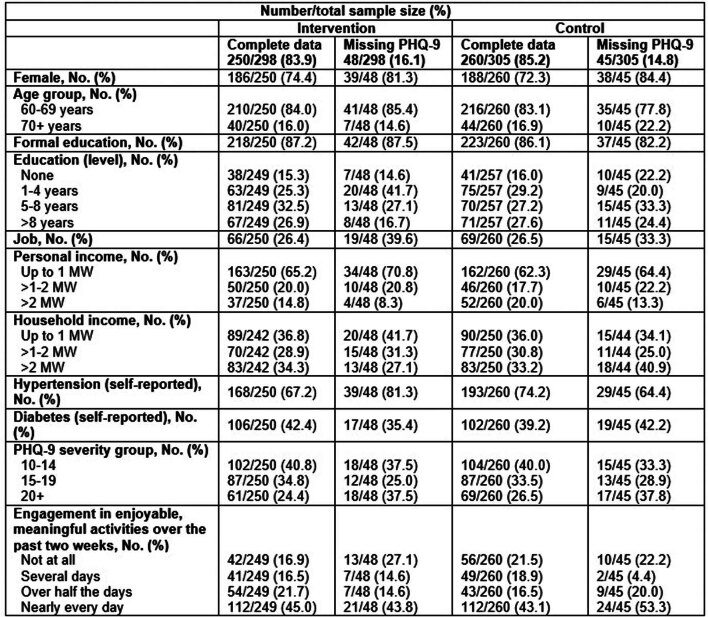
Comparison between participants with complete data and participants with missing data at the 5-month follow-up assessment

MW, minimum wage (in 2021, the minimum wage in Brazil was R$1,100 (approximately US$213)); PHQ-9, 9-item Patient Health Questionnaire.

**Extended Data Table 5:**
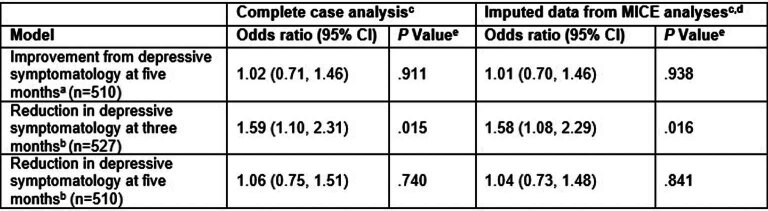
Comparison of adjusted estimates from multiple imputed data using the MICE models, with estimates from complete case analysis for the improvement from depressive symptomatology (PHQ-9 scores < 10) at 5 months, and for a reduction in PHQ-9 scores by at least 50% between baseline and 3 and 5 months (secondary outcome)

MICE, multiple imputation by chained equations; OR, odds ratio; PHQ-9, 9-item Patient Health Questionnaire

^a^The secondary outcome of improvement from depressive symptomatology was defined as PHQ-9 scores less than ten.

^b^The secondary outcome of reduction in depressive symptomatology was defined as PHQ-9 scores reducing by at least 50% between baseline and the first and second follow-up assessments (yes/no).

^c^Odds ratios and 95% CIs were calculated using logistic regression models adjusted for sex, age group and baseline severity of PHQ-9 (categorical).

^d^All estimates had missing data imputed by trial arm using MICE models that included sex, age group, baseline severity of PHQ-9 (categorical) and predictors of missingness.

^e^All *P* values reported are based on two-sided Wald tests from the relevant logistic regression model, with no adjustments for multiple tests.

**Extended Data Table 6:**
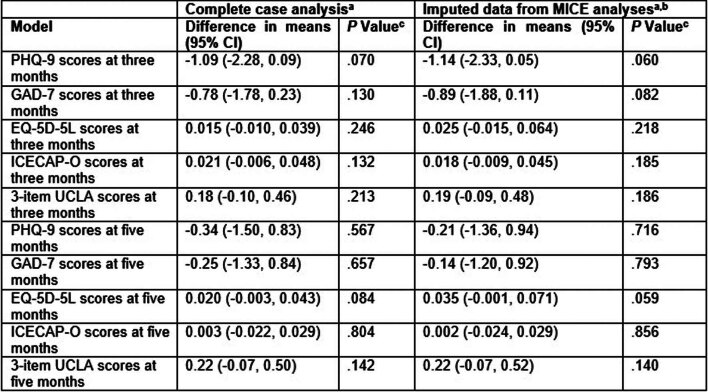
Comparison of estimates from multiple imputed data using MICE models, with estimates from complete case analysis for secondary outcomes at 3 and 5 months

CI, confidence interval; EQ-5D-5L, 5-level EuroQol health-related quality of life questionnaire; GAD-7, 7-item General Anxiety Disorder questionnaire; ICECAP-O, ICEpop CAPability measure for older people; PHQ-9, Patient Health Questionnaire-9; 3-item UCLA, University of California, Los Angeles.

PHQ-9 scores range from 0 to 27, with higher scores representing more severe depressive symptomatology.

GAD-7 scores range from 0 to 21, with higher scores representing more severe anxiety symptomatology.

EQ-5D-5L scores range from −0.264 to 1, with higher scores representing higher quality of life.

ICECAP-O scores range from 0 to 1, with higher scores representing greater levels of capability well-being.

3-item UCLA scores range from 3 to 9, with higher scores representing more severe levels of loneliness.

^a^Differences in means were estimated using linear regression models, adjusted for relevant baseline assessment of corresponding outcome, sex, age group and baseline severity of PHQ-9 (categorical).

^b^All estimates had missing data imputed seperately, according to trial arm, using MICE models that included predictors of missingness, sex, age group and baseline severity of the PHQ-9 score (categorical).

^c^All *P* values reported are based on two-sided *t*-tests from the relevant linear regression model, with no adjustments for multiple tests.

**Extended Data Table 7:**
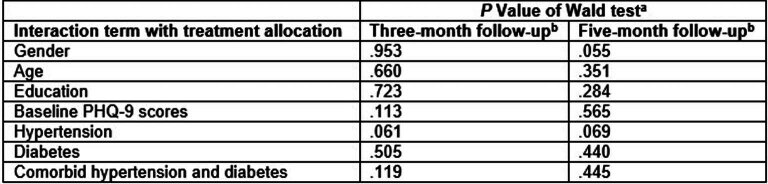
Results from subgroup analyses testing for the effect modification between prespecified variables and treatment arm at 3 and 5 months for the primary and secondary outcomes of improvement from depressive symptomatology using complete data only

OR, odds ratio; PHQ-9: 9-item Patient Health Questionnaire.

^a^All *P* values reported are based on two-sided Wald tests, with no adjustments for multiple tests.

^b^ORs and 95% CIs were calculated using logistic regression models adjusted for sex, age group and baseline severity of PHQ-9 (categorical).

**Extended Data Table 8:**
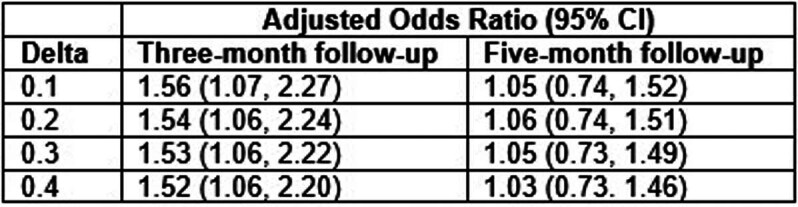
Adjusted ORs (95% CIs) for different departures from the missing at random assumption, for the outcome variable of improvement from depressive symptomatology (PHQ-9 scores < 10) at 3 and 5 months assuming greater probability of the outcome being missing when depression was present

CI, confidence interval; OR, odds ratio; PHQ-9, 9-item Patient Health Questionnaire.

## Data Availability

De-identified individual participant data and the data dictionary will be made available 24 months after publication. Proposals with specific aims and an analysis plan should be directed to the corresponding author (M.S.).

## Code availability

The code for the data analysis will also be made available 24 months after publication.
