# Supplementary material for: Self-help mobile messaging intervention for depression among older adults in resource-limited settings: a randomized controlled trial
Source: Nat Med. 2024 Mar 14;30(4):1127–33. doi: 10.1038/s41591-024-02864-4 (PMC11031393; doi:10.1038/s41591-024-02864-4)
Supplement: Supplementary file 1 — Trial protocol. [file 41591_2024_2864_MOESM1_ESM.pdf]

nature medicine

Article https://doi.org/10.1038/s41591-024-02864-4

Self-help mobile messaging intervention for depression among older adults in resource-limited settings: a randomized controlled trial

In the format provided by the authors and unedited

This Supplement contains the following items:

1 Original protocol

2 Final protocol

3 Summary of amendments

Digital psychosocial intervention for depression among older adults in socioeconomically deprived areas in Brazil (PRODIGITAL-D): protocol for an individually randomised controlled trial

Version 1.0

08 February 2021

Title (1)

"Digital psychosocial intervention for depression among older adults in socioeconomically deprived areas in Brazil (PRODIGITAL-D): protocol for an individually randomised controlled trial"

Trial registration (2a)

To be registered at Registro Brasileiro de Ensaios Clínicos (ReBEC).

Protocol version (3)

Version 1.0, 08 February 2021.

Funding (4)

Roles and responsibilities

Names and affiliations of protocol contributors (5a)

Name and contact information for the trial sponsor (5b)

Role of study sponsor and funders (5c)

The sponsors and funders have no roles in the design and conduction of the study, data analysis and interpretation, or in the writing or submission of the manuscript for publication.

Composition, roles, and responsibilities of the coordinating center and steering committee (5d)

Introduction

Background and rationale (6a)

Depression is a common but often neglected mental health disorder, especially among individuals living in socioeconomically vulnerable situations. Low- and middle-income countries are the most affected by this condition, where health systems are not able to meet high demands for mental health services due to scarce specialised healthcare professionals and economic resources (1, 2).The demographic changes that LMICs are experiencing add

another layer of concern, as it is known that depression at older ages has a high prevalence (2) and is associated with can increased healthcare utilisation and costs (3).

The World Health Organization (WHO) recommends the management of depression in primary healthcare, as it is affordable and cost-effective (4). Based on collaborative care and task sharing principles, our group developed the PROACTIVE intervention, a 17-week programme for the treatment of depression among older adults registered with primary care clinics in Brazil (5). Short, animated videos were shown during home sessions led by community health workers. The videos used psychoeducation and behavioural activation techniques to teach individuals to recognise their depressive symptoms, and to increase the number of meaningful and pleasant activities in their daily lives. PROACTIVE used a dedicated tablet computer application to structure intervention sessions, present multimedia content to trial participants, collect data, and allow communication between stakeholders (6). Due to the nature of the programme and our target population, the PROACTIVE home sessions were no longer feasible when the COVID-19 pandemic started. Therefore, we designed a remotely delivered intervention (Viva Vida) that will be evaluated in a separate individually randomised controlled trial (RCT) (PRODIGITAL-D) described in this protocol.

Alongside many challenges brought by the pandemic to primary care services, social distancing measures have also highlighted the need for developing evidence-based and cost-effective interventions that do not require primary care professionals or mental health specialists to conduct personal consultations. The development of digital health promotion programmes for mental health has become popular in recent years with the penetration of smartphones throughout the world (7). Evidence of the effectiveness of digital interventions for depressive symptoms is beginning to emerge from LMICs (8). Nevertheless, interventions targeting older populations have been less explored (9). We are therefore investigating a 12-week digital 'psychosocial' intervention delivered by automated WhatsApp messages to treat depression among older adults in Guarulhos, Brazil.

Explanation for the choice of comparators (6b)

Participants will receive a digital psychosocial intervention delivered through the WhatsApp application. The research team will not interfere with any other health care participants receive in either arm.

Objectives (7)

The objective of the PRODIGITAL-D study is to investigate the effectiveness and cost-effectiveness of treating older adults with depression by a 12-week digital psychosocial intervention (Viva Vida) delivered by WhatsApp messages, compared with a single message. We also aim to evaluate key implementation outcomes and how they influenced recovery from depression.

Trial design (8)

This is a two-arm, individually RCT with 1:1 allocation ratio and embedded economic and process evaluations.

Methods: Participants, interventions and outcomes

Study setting (9)

The study will recruit individuals registered with primary care clinics, known as Unidades Básicas de Saúde (UBS), in Guarulhos, Brazil. Guarulhos is the second most populous city of the state of São Paulo and marked by socioeconomic inequalities. The population is estimated to be 1.4 million, with 39 UBSs working to the Family Health Strategy model spread across four health districts. The 20 largest UBSs will participate in this study.

Eligibility criteria (10)

Inclusion criteria

- Individuals aged 60 or over
- Individuals registered with any of the participating UBSs
- Individuals able to receive and listen to WhatsApp messages
- Individuals screening positive for depressive symptomatology on the Patient Health Questionnaire (PHQ; scores of PHQ-2≥1 and PHQ-9≥10) (10)

Exclusion criteria

- Individuals with communication issues (non-Portuguese speaking, cognitively impaired or other problem hindering communication to engage in trial assessments or intervention, such as vision or hearing problems)
- Individuals unable to engage in the study for the total period of five months (terminal illness or partner with terminal illness, other)

- Individuals presenting acute suicidal risk (i.e. reported suicidal attempt in the two weeks prior to the screening assessment, as assessed by the 9th item of the PHQ-9 and the Immediate Suicide Risk Protocol)
- Individuals living in the same household as another participant in the study
- Individuals who participated in the PROACTIVE trial

Interventions (11a)

Digital psychosocial intervention

Participants allocated to the intervention arm will receive the 12-week Viva Vida digital psychosocial programme delivered via WhatsApp messages. The contents of this programme are based on the PROACTIVE intervention (5) and will include psychoeducation about depression and health promotion guidelines, simple ways to solve day-to-day problems related to depressive symptoms, and behaviour activation. Such an approach is anchored in recommendations by the WHO in guides containing step-by-step orientation for digital interventions targeting depression (11). The proposed intervention is also based on concepts of interactive applications of health communication (12, 13). Participants will receive audio and visual messages, delivered four days a week in the morning and afternoon over a 12-week period.

The audio messages have on average three minutes and use the technique of storytelling, which is a powerful communication tool when used to share and create bonds with other individuals. This technique can involve, convince, remind, and motivate. It can also improve the attention and retention of important information by the target audience. By creating empathy and interest in a story, we can encourage new behaviours and reflections on personal problems.

Once a week, participants will be invited to share their opinions about the programme by responding to a question through the WhatsApp ‘quick reply’ tool, with a up to three-answer option (e.g. yes, more or less, no). After answering the question, participants will also be invited to record and send audio messages to share their experience with the programme. Messages sent by the participants will be answered by automated reply only, as this programme was not designed to reply to individual demands. However, during the intervention, participants will receive messages advising them that they can visit health professionals to receive further advice and care for depression if they feel they need additional support.

Single message

Participants allocated to the control group will receive a single audio message with psychosocial contents about depression, such as the main signs of depression and simple ways to improve their mood. Participants will also be advised to talk to health professionals if they do not feel better.

System to deliver the messages

During the PROACTIVE RCT, 68% of 2,246 screened older adults who owned a mobile phone reported sending and/or receiving messages by WhatsApp. Thus, considering the WhatsApp is the most used messaging application in Brazil and it is user-friendly for older adults, a web system integrated into the WhatsApp Business API (Application Programming Interface) will be developed to deliver the messages for PRODIGITAL-D participants. The WhatsApp Business API enables us to manage and deliver messages to a large number of participants and to collect information, such as date and time when each message was delivered and visualised, timestamp and content of incoming messages, and participants' interaction with WhatsApp features (e.g. the aforementioned quick replies).

An intermediate company's platform will be used to register templates of the messages to be sent during the intervention. These templates are approved by the WhatsApp platform prior to first use. They allow messages to be personalised with the name of each participant, the attachment of appropriate media file (audio/image) and other automation processes.

Messages will be scheduled weekly according to the lists of randomised participants and delivered using a 'cron service' (job scheduler) on the server, in which commands or scripts are set to run periodically at fixed times, dates or intervals.

Criteria for discontinuing or modifying allocated interventions (11b)

After the first two weeks of receiving the Viva Vida programme (see section 11c), participants identified with a communication pattern that might indicate that our WhatsApp number was blocked (consecutive WhatsApp messages not delivered to participants) will be removed from our broadcast list to abide by WhatsApp policy and to keep our system functional. No new intervention messages will be sent, but participants will be contacted for the follow-up assessment, unless they withdraw their consent for follow up.

Strategies to improve adherence to interventions (11c)

During the first two weeks participants receive the intervention, the research team will actively identify those who might have an issue receiving or opening the messages, based on the status of the WhatsApp messages, and contact them by phone. After this period, no other strategies will be adopted. Participants who have technical difficulties using the application will be able to contact a dedicated phone number to try to solve the issue with support from the research team throughout the whole period of the trial.

Relevant concomitant care permitted or prohibited during the trial (11d)

Neither ongoing nor new pharmacological or non-pharmacological treatments, nor health appointments, will be prohibited during the trial.

Outcomes (12)

Primary outcome

We will assess the proportion of participants recovered from depression with the PHQ-9 (14, 15) (PHQ-9 score<10), four months after receiving the first message (intervention group) or the single message (control group).

Secondary outcomes

The proportion recovered at eight months (PHQ-9<10) will assess the maintenance of any earlier clinical gains. The continuous score of the PHQ-9 will also be considered at four and eight months, as well as, anxiety symptomatology assessed with the Generalized Anxiety Disorder-7 (GAD-7) (16) and loneliness assessed with the 3-item University of California, Los Angeles (UCLA) loneliness scale (3-item UCLA) (17).

Cost-effectiveness outcomes

Assessment of quality of life with the European Quality of Life five-dimensional questionnaire, five-level version (EQ-5D-5L) (18), and capability wellbeing with the ICEpop CAPability measure for Older people (ICECAP-O) (19) at four and eight months.

Participant timeline (13)

Participants will be randomised after granting consent to participate in the trial and will be included in a list to start receiving the messages (both control and intervention arms) no more than 10 days after consenting to participate. Each list will include participants recruited during the previous week who will receive the single message (control group) or the first message (intervention group) on the following Monday. Participants allocated to the

intervention arm will receive messages every Monday, Wednesday, Friday and Saturday twice a day during 12 weeks. The follow-up assessment will occur at four and eight months after receiving the first message. Figure 1 shows the complete timeline of the study.

Sample size (14)

A sample size of 330-374 individuals will detect a 15 percentage point difference (25% versus 40%) in recovery between the control and intervention groups after three months, with 80-85% power and two-sided 5% significance level. Such a difference in recovery rate is considered clinically meaningful (20-24). We anticipate 25% attrition, which is readily achievable according to the experience we had collecting follow-up data by phone in our previous RCT in Guarulhos, yielding a corrected total sample size of 440-500.

All of the following estimates are based on the PROACTIVE RCT (5) and our pilot study in São Paulo (25). We expect that on average 10% of the individuals registered with each UBS will be aged 60 years or older. On average, there are approximately 1,600 individuals in the eligible age range registered with each UBS. Based on the PROACTIVE data, we would be able to reach 440 individuals by phone and 60% of them (250 individuals) would have a mobile phone and would use WhatsApp application. With a proportion of depressive symptomatology (PHQ-9≥10) of around 20% (25), there would be 50 individuals potentially eligible in each UBS, though this is likely to reduce to 35 once other entry criteria are applied.

We will work with at least 24 UBSs, and we plan to complete recruitment in six months. We will need to screen approximately 25,000 individuals to achieve a sample of 500 participants. Of these 25,000 individuals, we expect that we will be able to contact 6,800. Among the individuals we can contact, 3,800 will use WhatsApp. We expect that at least 20% will have depressive symptomatology (n=760), and 530 will consent to participate in the study – that is, at the upper end of the above range from our sample size calculation.

Recruitment (15)

A list with names and contact details of all individuals aged 59 years or over and registered within the 24 UBSs will be provided by the Health Secretariat of Guarulhos. We will exclude duplicated names and PROACTIVE participants from the list. All individuals in the list will receive a random ID. These IDs will be ordered before entering the study system and the recruitment will follow this order. We will first approach individuals by sending WhatsApp messages to those who have at least one mobile phone number. The message will give a

brief introduction to the study and will inform individuals that a research assistant will contact them soon. Initially, only those whose WhatsApp message was successfully delivered will be contacted.

The recruitment comprises three parts: (a) screening; (b) baseline; and (c) invitation to participate in the study. Screening and baseline assessment will be carried out consecutively where individuals will be invited to participate in the study during the same phone call, whenever it is possible. The following are details of each stage of the recruitment process:

a. Screening: assessment of inclusion criteria. Before starting the screening, we will inform the participant about the interview and seek consent. Then, we will confirm the age and the use of WhatsApp. Individuals will be screened with the first two questions of the PHQ-9 (PHQ-2). If they present at least one core symptom of depression (depression mood and anhedonia, i.e. PHQ-2≥1) (26), the evaluation of depressive symptomatology will be completed with the remaining seven questions (10).

b. Baseline: Older adults who use WhatsApp, with PHQ-9≥10 (cut-off for depressive symptomatology) (10) and who do not present acute suicidal risk (as assessed by the 9th item of the PHQ-9 and the Immediate Suicide Risk Protocol) or any other exclusion criteria will complete a full baseline assessment.

c. Invitation to participate in the clinical trial: after completing the baseline assessment, the research assistant will provide information about the study (type of interventions and follow-up assessments) for all eligible individuals and invite them to participate in the study. Additionally, the WhatsApp number will be 'revalidated' by sending and confirming the receipt of a 'Welcome' message.

Assignment of interventions: allocation

Sequence generation (16a)

Participants will be randomly allocated in a 1:1 ratio to either intervention or control arms. Stratification will be based on the PHQ-9 groups (scores of 10-14, 15-19 and 20+), gender (male and female) and age groups (60-69, 70-79 and 80+ years). Random permuted blocks with random block sizes will be used to generate the randomisation sequence with the support of Microsoft Excel.

Concealment mechanism (16b)

The randomisation sequence will be concealed in the "randomization module" of the Research Electronic Data Capture (REDCap).

Implementation (16c)

The allocation sequence will be generated by members of the research team (CAN and TJP) not directly involved in recruiting individual participants, who will be enrolled by an independent research assistant. Data collected will be reviewed by the research coordinator after the participant's consent to participate in the study and the study arm will be allocated with the support of REDCap. The intervention team will receive the list of randomised participants and will send the allocated messages.

Assignment of interventions: Blinding

Who will be blinded (17a)

Given the difference between the number and contents of the messages received by the intervention and control groups, blinding of participants will not be feasible. A list with the participants' allocation group will be sent every week to the researchers responsible for sending the messages. Research assistants will be completely blind to the group allocation during the follow-up assessments and, whenever possible, the research assistant will not carry out more than one assessment (recruitment and follow-ups) with the same participant. The research teams that coordinate the recruitment and intervention will work separately. The assessments and the interventions will be delivered remotely and individually in order to minimise the risk of contamination.

Procedure for unblinding if needed (17b)

No procedure for unblinding is planned for this trial.

Data collection and management

Plans for assessment and collection of outcomes (18a)

Recruitment and the 4- and 8-month follow-up interviews will be carried out by independent research assistants through phone calls. The research assistants will be trained to conduct the assessments and to use the data collection software (REDCap). Quality control of a sample of recorded assessments will be conducted by an independent research assistant. Qualitative assessments will be carried out while participants are receiving the intervention and after the first follow-up assessment.

Instruments

Depressive symptomatology will be assessed using the PHQ-9 (10). This instrument was chosen as it is widely used and there is evidence of validity in Brazil (27). Anxiety symptomatology will be initially screened with the first two questions of the GAD-7 (16) and if GAD-2≥1 (28), the remaining five questions will be assessed. The 3-item UCLA will be used to assess loneliness (17). Quality of Life will be assessed with the EQ-5D-5L (18) and the ICECAP-O (19). Level of depressive symptomatology was associated with these two measures in our previous study (data not published). Physical and chronic conditions, depression treatment, sociodemographic profile (level of education, personal and household income, marital status, race and living arrangement), alcohol and tobacco use, and use of WhatsApp will also be asked during the recruitment assessment.

During both follow-up assessments, questionnaires completed at baseline will be repeated including the PHQ-9, GAD-7, 3-item UCLA, EQ-5D-5L and ICECAP-O. Participants will also be asked about admissions to hospital and consultations related to mental healthcare and any ongoing treatment for depression. Additionally, questions about the experience receiving the messages will be included for the intervention arm. For every participant, we will request the electronic health record system data on psychotropic medications and consultations with doctors and nurses during the period they were participating in the trial.

Plans to promote participant retention and complete follow-up (18b)

For each follow-up, we will adopt a 4-week window in order to maximise our chances to complete the follow-up assessments. Whenever we have issues with contacting the participant by phone call, we will first send him/her an audio and/or a text message by WhatsApp with a reminder about the assessment. If the participant does not reply to these messages, the UBS manager or an appointed staff member will be contacted to help us reach him/her. If we are unable to find the participant by phone within the first three weeks of the follow-up window, a research assistant will attempt to visit the participant at home to complete a face-to-face assessment.

Data management (19)

All assessment data will be collected and managed using REDCap (29, 30) hosted at the Hospital das Clínicas da Faculdade de Medicina da Universidade de Sao Paulo. REDCap is a secure, web-based software platform designed to support data capture for research studies, providing: 1) an intuitive interface for validated data capture; 2) audit trails for

tracking data manipulation and export procedures; 3) automated export procedures for seamless data downloads to common statistical packages; and 4) procedures for data integration and interoperability with external sources.

Statistical methods

Statistical methods for primary and secondary outcomes (20a)

The analysis for all outcome measures will follow the intention-to-treat principle and adhere to Consolidated Standards of Reporting Trials guidelines for randomised trials (31). To understand if there are any imbalances in risk factors for depression, descriptive statistics will be used to compare differences in baseline characteristics between treatment arms.

All outcomes will be evaluated using regression models that adjust for stratification, the baseline assessment of the corresponding outcome measure. For secondary analyses only, any baseline variables that are not balanced between treatment arms will be accounted for in these models. Logistic regression models will be used to evaluate for the primary outcome (recovery from depression at four months) and the secondary outcome (recovery from depression at eight months). Odds ratios with 95% confidence intervals will be used to estimate recovery from depression at four and eight months. Other secondary outcomes including PHQ-9, GAD-7, 3-item UCLA, EQ-5D-5L and ICECAP-O scores will be assessed using linear regression models. Coefficients and associated 95% confidence intervals for these outcomes represent differences in means between the intervention and control arms. Sensitivity analyses will be conducted to investigate any potential clustering at the USB level.

Economic analysis

Two economic analyses will be conducted. 1) Incremental cost-effectiveness between the two arms of the trial will be estimated using: a) the primary clinical outcome measure (cost per patient recovered); and b) Quality Adjusted Life Years (QALYs) calculated using the EQ-5D-5L (32, 33). These results will be presented as incremental cost-effectiveness ratios and cost-effectiveness acceptability curves. These show the probability of the intervention being cost-effective at a range of 'willingness-to-pay' threshold levels. 2) The net monetary benefit statistic (NMB), using the difference in costs and the difference in QALYs between the two arms, will be calculated at the WHO recommended threshold for LMICs.

We will conduct the cost-effectiveness analysis from the health system perspective to compare the costs and effects of the 12-week digital psychosocial programme against a single message. The EQ-5D-5L (32, 34, 35) responses will be converted into utility scores using the population tariff most appropriate for the Brazilian population available at the time of the analysis. Utility scores will be used to estimate QALYs, adjusting for baseline values. We will use national, where available, or local unit costs to value resource use.

Methods for additional analyses (e.g. subgroup analyses) (20b)

A Complier Average Case Effect (CACE) analysis will be used to determine the effect of number of messages listened to on recovery from depression at both four and eight months. A threshold value (listening to most of the messages received) will be used for the CACE analysis with PHQ-9 scores at both time points. We will use this threshold as it was hypothesised to be the minimum number of sessions needed to have a therapeutic effect. A sensitivity analysis using the thresholds of listening to at least half of the messages or all messages will be conducted, and we will also consider using the number of messages opened to as a continuous variable unless the relevant relationship is far from linear.

Additional exploratory subgroup analyses will be used to estimate whether the following pre-specified characteristics of the participants modified the effect of the intervention: baseline PHQ-9; gender; age; education level; presence of co-morbid physical illnesses. To test for modification, an interaction term will be introduced between the pre-specified variable and the treatment arm. Results of these analyses will need to be interpreted with caution due to the limited power to detect such interactions as well as the paucity of evidence on the theoretical basis for these hypotheses.

Methods in analysis to handle protocol non-adherence and any statistical methods to handle missing data (20c)

Patterns and the proportion of missing data for the primary and secondary outcomes will be investigated. If there are any marked differences in missing data between treatment arms, or if greater than 10% of the data are missing, multiple imputation using chained equations (MICE models) under the assumption that data is missing at random (MAR) will be used. Data will be imputed separately for the different treatment arms and MICE models will include any variables that predict missingness. Sensitivity analyses testing for modest departures against the MAR assumption will be conducted using the Selection Model

Approach.

Composition of the Data Monitoring Committee, its role and reporting structure (21a)

Data Monitoring Committee (DMC)

Under the guidance of the TSC, a Data Monitoring Committee (DMC) will be formed comprising an independent Chair, an independent statistician, one other independent member, and at least one of the trial statisticians.

Interim analyses (21b)

No interim analyses are planned for this trial.

Adverse event reporting and harms (22)

The risk associated with the trial and the digital intervention are considered minimal. Participants will receive any pharmacological and non-pharmacological treatment they were prescribed before or during the study. Also, participants of both arms will receive messages advising them to seek healthcare if the symptoms are not improving or if they are worsening. Identification of acute suicide risk at recruitment or follow-up assessments will activate a standardised protocol, and a family member and the UBS manager will be contacted by the research team. This protocol was successfully applied in the PROACTIVE study (5, 25). Individuals identified with an acute suicide risk during the recruitment assessment will not be eligible to participate in the study.

Frequency and plans for auditing trial conduct (23)

Characteristics used for stratification of randomisation will be monitored in order to ensure that the allocation sequence and REDCap “randomization module” are working as expected, and the two study arms are reasonably well balanced in each stratum. The TSC will be presented with a report of the trial conduct.

Ethics approval and consent to participate (24)

Ethical approval was submitted to the Ethics Committee of the Hospital das Clínicas da Faculdade de Medicina da Universidade de Sao Paulo – HCFMUSP (CAPPesq, ref: 4.097.596). This study was authorised by the Secretaria da Saúde do Município de Guarulhos.

Plans for communicating important protocol amendments to relevant parties (25)

Modifications on the study protocol will be discussed between the research team, TSC and DMC and submitted to approval of the Ethics Committee. Whenever relevant, Guarulhos health system managers and coordinators will be consulted before any decision.

Who will take informed consent? (26a)

Verbal informed consent will be sought by the research assistant before screening and qualitative assessments and when inviting the eligible individuals to participate in the study. All assessments will be conducted by phone. Verbal consent and assessments will be recorded whenever authorised by the individual.

Additional consent provisions for collection and use of participant data and biological specimens (26b)

No additional consent for collection and use of participant data and biological specimens will be collected.

Confidentiality (27)

This study will be compliant to the National Commission of Ethics in Research (Comissão Nacional de Ética em Pesquisa), Resolution 466/2012 security policies. One member of the team will extract the individual's information from the list provided by the Health Secretariat of Guarulhos, and then they will receive a random ID number. The same ID number will be used when entering data into the REDCap platform and the system developed to deliver the messages. The access to both systems is secured by password and limited according to the members of the research team's assigned roles and permissions. Audio files of recruitment, follow-up and qualitative assessments will be renamed using the corresponding ID number and initials and stored on a password-protected platform. Only the recruitment coordination team will have access to these audio files. No paper records will be kept.

Declaration of interests (28)

The authors have no conflict of interest to declare.

Access to data (29)

Study documentation and trial data will be available on request according to data sharing conditions.

Ancillary and post-trial care (30)

No post-trial care is planned for this study.

Dissemination policy (31a)

Publications on internationally competitive journals and presentation at relevant conferences are expected from this trial. Meetings with the main stakeholders will be organised at the end of the trial to present the main results and discuss further steps and potential future collaboration.

Authors' contributions (31b)

Plans to give access to the full protocol, participant level-data and statistical code (31c)

Participant level-data and statistical code will be available for external use 24 months after publishing the paper with effectiveness results. A research proposal with defined aims and a statistical analysis plan should accompany any request and all requests will be evaluated by the joint principal investigators before granting access to the data.

Consent for publication (32)

Participants will be advised that non-identifiable data will be used for publications before granting consent in both assessments.

Plans for collection, laboratory evaluation and storage of biological specimens for genetic or molecular analysis in this trial/future use (33)

No biological specimens will be collected during this study.

References

1. Rathod S, Pinninti N, Irfan M, Gorczynski P, Rathod P, Gega L, et al. Mental Health Service Provision in Low- and Middle-Income Countries. Health Serv Insights. 2017;10:1178632917694350.
2. World Health Organization. Depression and Other Common Mental Disorders. Global Health Estimates.2017. Available from: http://apps.who.int/iris/bitstream/handle/10665/254610/WHO-MSD-MER-2017.2-eng.pdf.
3. World Health Organization. Mental health of older adults 2017 [Available from: https://www.who.int/news-room/fact-sheets/detail/mental-health-of-older-adults.
4. World Federation for Mental Health. Depression: a global crisis. World Mental Health Day, October 10 20122012. Available from: https://www.researchgate.net/publication/285075782_Depression_A_global_public_health_concern
5. Scazufca M, Nakamura CA, Peters TJ, Henrique MG, Seabra A, La Rotta EG, et al. A collaborative care psychosocial intervention to improve late life depression in socioeconomically deprived areas of Guarulhos, Brazil: the PROACTIVE cluster randomised controlled trial protocol. Trials. 2020;21(1):914.
6. Van de Ven P, Araya R, P de Paula Couto MC, Henrique MG, Meere D, Vilela Mendes A, et al. Investigating Software Requirements for Systems Supporting Task-Shifted Interventions: Usability Study. J Med Internet Res. 2019;21(11):e11346.
7. Firth J, Torous J, Nicholas J, Carney R, Pratap A, Rosenbaum S, et al. The efficacy of smartphone-based mental health interventions for depressive symptoms: a meta-analysis of randomized controlled trials. World Psychiatry. 2017;16(3):287-98.
8. Fu Z, Burger H, Arjadi R, Bockting CLH. Effectiveness of digital psychological interventions for mental health problems in low-income and middle-income countries: a systematic review and meta-analysis. Lancet Psychiatry. 2020;7(10):851-64.
9. Riadi I, Kervin L, Teo K, Churchill R, Cosco TD. Digital Interventions for Depression and Anxiety in Older Adults: Protocol for a Systematic Review. JMIR Res Protoc. 2020;9(12):e22738.
10. Kroenke K, Spitzer RL, Williams JB. The PHQ-9: validity of a brief depression severity measure. J Gen Intern Med. 2001;16(9):606-13.
11. Carswell K, Harper-Shehadeh M, Watts S, Van't Hof E, Abi Ramia J, Heim E, et al. Step-by-Step: a new WHO digital mental health intervention for depression. Mhealth. 2018;4:34.
12. Mehrotra S, Tripathi R. Recent developments in the use of smartphone interventions for mental health. Curr Opin Psychiatry. 2018;31(5):379-88.
13. Chen YR, Schulz PJ. The Effect of Information Communication Technology Interventions on Reducing Social Isolation in the Elderly: A Systematic Review. J Med Internet Res. 2016;18(1):e18.
14. Lowe B, Unutzer J, Callahan CM, Perkins AJ, Kroenke K. Monitoring depression treatment outcomes with the patient health questionnaire-9. MedCare. 2004;42(12):1194-201.
15. Kroenke K, Spitzer RL, Williams JB. The PHQ-9: validity of a brief depression severity measure. JGenInternMed. 2001;16(9):606-13.
16. Spitzer RL, Kroenke K, Williams JB, Löwe B. A brief measure for assessing generalized anxiety disorder: the GAD-7. Arch Intern Med. 2006;166(10):1092-7.
17. Hughes ME, Waite LJ, Hawkley LC, Cacioppo JT. A Short Scale for Measuring Loneliness in Large Surveys: Results From Two Population-Based Studies. Res Aging. 2004;26(6):655-72.
18. Devlin NJ, Krabbe PF. The development of new research methods for the valuation of EQ-5D-5L. Eur J Health Econ. 2013;14 Suppl 1:S1-3.
19. Grewal I, Lewis J, Flynn T, Brown J, Bond J, Coast J. Developing attributes for a generic quality of life measure for older people: preferences or capabilities? Soc Sci Med. 2006;62(8):1891-901.
20. Ciechanowski P, Wagner E, Schmaling K, Schwartz S, Williams B, Diehr P, et al. Community-integrated home-based depression treatment in older adults: a randomized controlled trial. JAMA. 2004;291(13):1569-77.
21. Araya R, Rojas G, Fritsch R, Gaete J, Rojas M, Simon G, et al. Treating depression in primary care in low-income women in Santiago, Chile: a randomised controlled trial. Lancet. 2003;361(9362):995-1000.

22. Unutzer J, Katon W, Callahan CM, Williams JW, Jr., Hunkeler E, Harpole L, et al. Collaborative care management of late-life depression in the primary care setting: a randomized controlled trial. JAMA. 2002;288(22):2836-45.
23. Cramer H, Salisbury C, Conrad J, Eldred J, Araya R. Group cognitive behavioural therapy for women with depression: pilot and feasibility study for a randomised controlled trial using mixed methods. BMC Psychiatry. 2011;13.
24. Richards DA, Hill JJ, Gask L, Lovell K, Chew-Graham C, Bower P, et al. Clinical effectiveness of collaborative care for depression in UK primary care (CADET): cluster randomised controlled trial. BMJ. 2013;347:f4913.
25. Scazufca M, de Paula Couto MCP, Henrique MG, Mendes AV, Matijasevich A, Pereda PC, et al. Pilot study of a two-arm non-randomized controlled cluster trial of a psychosocial intervention to improve late life depression in socioeconomically deprived areas of São Paulo, Brazil (PROACTIVE): feasibility study of a psychosocial intervention for late life depression in São Paulo. BMC Public Health. 2019;19(1):1152.
26. Kroenke K, Spitzer RL, Williams JB. The Patient Health Questionnaire-2: validity of a two-item depression screener. Med Care. 2003;41(11):1284-92.
27. Santos IS, Tavares BF, Munhoz TN, Almeida LS, Silva NT, Tams BD, et al. [Sensitivity and specificity of the Patient Health Questionnaire-9 (PHQ-9) among adults from the general population]. Cad Saude Publica. 2013;29(8):1533-43.
28. Kroenke K, Spitzer RL, Williams JB, Monahan PO, Löwe B. Anxiety disorders in primary care: prevalence, impairment, comorbidity, and detection. Ann Intern Med. 2007;146(5):317-25.
29. Harris PA, Taylor R, Thielke R, Payne J, Gonzalez N, Conde JG. Research electronic data capture (REDCap)--a metadata-driven methodology and workflow process for providing translational research informatics support. J Biomed Inform. 2009;42(2):377-81.
30. Harris PA, Taylor R, Minor BL, Elliott V, Fernandez M, O'Neal L, et al. The REDCap consortium: Building an international community of software platform partners. J Biomed Inform. 2019;95:103208.
31. Schulz KF, Altman DG, Moher D, Group C. CONSORT 2010 Statement: updated guidelines for reporting parallel group randomised trials. Trials. 2010;11:32.
32. Williams A, Kind P. The present state of play about QALYs. In: Hopkins A, editor. Measures of the quality of life and the uses to which such measures may be put. London: RCP Publications; 1992.
33. Devlin NJ, Krabbe PF. The development of new research methods for the valuation of EQ-5D-5L. The European journal of health economics : HEPAC : health economics in prevention and care. 2013;14:S1-S3.
34. Sapin C, Fantino B, Nowicki ML, Kind P. Usefulness of EQ-5D in Assessing Health Status in Primary Care Patients with Major Depressive Disorder. Health and Quality of Life Outcomes. 2004;2:20.
35. Viegas Andrade M, Noronha K, Kind P, Maia AC, Miranda de Menezes R, De Barros Reis C, et al. Societal Preferences for EQ-5D Health States from a Brazilian Population Survey. Value in Health Regional Issues. 2013;2:405-12.

Digital psychosocial intervention for depression among older adults in socioeconomically deprived areas in Brazil (PRODIGITAL-D): protocol for an individually randomised controlled trial

Version 5.0

16 March 2022

Title (1)

"Digital psychosocial intervention for depression among older adults in socioeconomically deprived areas in Brazil (PRODIGITAL-D): protocol for an individually randomised controlled trial"

Trial registration (2a)

Registro Brasileiro de Ensaios Clínicos (ReBEC), RBR-4c94dtn. Registered on 22 October 2021 (submitted on 03 August 2021).

Protocol version (3)

Version 5.0, 16 March 2022.

Funding (4)

Roles and responsibilities

Names and affiliations of protocol contributors (5a)

Name and contact information for the trial sponsor (5b)

Role of study sponsor and funders (5c)

The sponsors and funders have no roles in the design and conduction of the study, data analysis and interpretation, or in the writing or submission of the manuscript for publication.

Composition, roles, and responsibilities of the coordinating center and steering committee (5d)

Introduction

Background and rationale (6a)

Depression is a common but often neglected mental health disorder, especially among individuals living in socioeconomically vulnerable situations. Low- and middle-income countries are the most affected by this condition, where health systems are not able to meet high demands for mental health services due to scarce specialised healthcare professionals and economic resources (1, 2).The demographic changes that LMICs are experiencing add

another layer of concern, as it is known that depression at older ages has a high prevalence (2) and is associated with can increased healthcare utilisation and costs (3).

The World Health Organization (WHO) recommends the management of depression in primary healthcare, as it is affordable and cost-effective (4). Based on collaborative care and task sharing principles, our group developed the PROACTIVE intervention, a 17-week programme for the treatment of depression among older adults registered with primary care clinics in Brazil (5). Short, animated videos were shown during home sessions led by community health workers. The videos used psychoeducation and behavioural activation techniques to teach individuals to recognise their depressive symptoms, and to increase the number of meaningful and pleasant activities in their daily lives. PROACTIVE used a dedicated tablet computer application to structure intervention sessions, present multimedia content to trial participants, collect data, and allow communication between stakeholders (6). Due to the nature of the programme and our target population, the PROACTIVE home sessions were no longer feasible when the COVID-19 pandemic started. Therefore, we designed a remotely delivered intervention (Viva Vida) that will be evaluated in a separate individually randomised controlled trial (RCT) (PRODIGITAL-D) described in this protocol.

Alongside many challenges brought by the pandemic to primary care services, social distancing measures have also highlighted the need for developing evidence-based and cost-effective interventions that do not require primary care professionals or mental health specialists to conduct personal consultations. The development of digital health promotion programmes for mental health has become popular in recent years with the penetration of smartphones throughout the world (7). Evidence of the effectiveness of digital interventions for depressive symptoms is beginning to emerge from LMICs (8), including Brazil (9). Nevertheless, interventions targeting older populations have been less explored (10). We are therefore investigating a 6-week digital ‘psychosocial’ intervention delivered by automated WhatsApp messages to treat depression among older adults in Guarulhos, Brazil.

Explanation for the choice of comparators (6b)

Participants will receive a digital psychosocial intervention delivered through the WhatsApp application. The research team will not interfere with any other health care participants receive in either arm.

Objectives (7)

The objective of the PRODIGITAL-D study is to investigate the effectiveness and cost-effectiveness of treating older adults with depression by a 6-week digital psychosocial intervention (Viva Vida) delivered by WhatsApp messages, compared with a single message. We also aim to evaluate key implementation outcomes and how they influenced recovery from depression.

Trial design (8)

This is a two-arm, individually RCT with 1:1 allocation ratio and embedded economic and process evaluations.

Methods: Participants, interventions and outcomes

Study setting (9)

The study will recruit individuals registered with primary care clinics, known as Unidades Básicas de Saúde (UBS), in Guarulhos, Brazil. Guarulhos is the second most populous city of the state of São Paulo and marked by socioeconomic inequalities. The population is estimated to be 1.4 million, with 39 UBSs working to the Family Health Strategy model spread across four health districts. The 24 largest UBSs will participate in this study.

Eligibility criteria (10)

Inclusion criteria

- Individuals aged 60 or over
- Individuals registered with any of the participating UBSs
- Individuals able to receive and listen to WhatsApp messages
- Individuals screening positive for depressive symptomatology on the Patient Health Questionnaire (PHQ; scores of PHQ-2≥1 and PHQ-9≥10) (11)

Exclusion criteria

- Individuals with communication issues (non-Portuguese speaking, cognitively impaired or other problem hindering communication to engage in trial assessments or intervention, such as vision or hearing problems)
- Individuals unable to engage in the study for the total period of five months (terminal illness or partner with terminal illness, other)

- Individuals presenting acute suicidal risk (i.e. reported suicidal attempt in the two weeks prior to the screening assessment, as assessed by the 9th item of the PHQ-9 and the Immediate Suicide Risk Protocol)
- Individuals living in the same household as another participant in the study
- Individuals who participated in the PROACTIVE trial

Interventions (11a)

Digital psychosocial intervention

Participants allocated to the intervention arm will receive the 6-week Viva Vida digital psychosocial programme delivered via WhatsApp messages. The contents of this programme are based on the PROACTIVE intervention (5) and will include psychoeducation about depression and health promotion guidelines, simple ways to solve day-to-day problems related to depressive symptoms, and behaviour activation. Such an approach is anchored in recommendations by the WHO in guides containing step-by-step orientation for digital interventions targeting depression (12). The proposed intervention is also based on concepts of interactive applications of health communication (13, 14). Participants will receive approximately 48 audio and visual messages, delivered four days a week in the morning and afternoon over a 6-week period. The duration of six weeks was chosen as an appropriate timeframe to ensure sustained participation throughout the whole period.

The audio messages have on average three minutes and use the technique of storytelling, which is a powerful communication tool when used to share and create bonds with other individuals. This technique can involve, convince, remind, and motivate. It can also improve the attention and retention of important information by the target audience. By creating empathy and interest in a story, we can encourage new behaviours and reflections on personal problems.

Once a week, participants will be invited to share their opinions about the programme by responding to a question through the WhatsApp ‘quick reply’ tool, with a up to three-answer option (e.g. yes, more or less, no). After answering the question, participants will also be invited to record and send audio messages to share their experience with the programme. Messages sent by the participants will be answered by automated reply only, as this programme was not designed to reply to individual demands. However, during the intervention, participants will receive messages advising them that they can visit health professionals to receive further advice and care for depression if they feel they need additional support.

Single message

Participants allocated to the control group will receive a single audio message with psychosocial contents about depression, such as the main signs of depression and simple ways to improve their mood. Participants will also be advised to talk to health professionals if they do not feel better.

System to deliver the messages

During the PROACTIVE RCT, 68% of 2,246 screened older adults who owned a mobile phone reported sending and/or receiving messages by WhatsApp. Thus, considering the WhatsApp is the most used messaging application in Brazil and it is user-friendly for older adults, a web system integrated into the WhatsApp Business API (Application Programming Interface) was developed to deliver the messages for PRODIGITAL-D participants. The PHP-based Laravel Framework was used in a Linux environment to create the web system, with Apache used to implement the server component and the data retained in a MySQL database. The system is hosted on a cloud service (Cloudways) where the access is restricted using both authentication and authorisation processes. Data flowing to and from browsers are also protected by the use of encryption through Secure Socket Layer certificates. An intermediate company was contracted, as the direct usage of the WhatsApp Business API is restricted to medium and large businesses. The WhatsApp Business API enables us to manage and deliver messages to a large number of participants and to collect information, such as date and time when each message was delivered and visualised, timestamp and content of incoming messages, and participants' interaction with WhatsApp features (e.g. the aforementioned quick replies).

The intermediate company's platform is used to register templates of the messages to be sent during the intervention. These templates are approved by the WhatsApp platform prior to first use. They allow messages to be personalised with the name of each participant, the attachment of appropriate media file (audio/image) and other automation processes.

Messages will be scheduled weekly according to the lists of randomised participants and delivered using a 'cron service' (job scheduler) on the server, in which commands or scripts are set to run periodically at fixed times, dates or intervals. A dashboard was also developed to track the communication pattern (that is, status of the WhatsApp messages – sent to the intermediate company's platform, delivered to participants, and opened by participants) and

the general performance of the scheduled lists to help us in identifying and following up participants with potential technical problems.

Criteria for discontinuing or modifying allocated interventions (11b)

After the first two weeks of receiving the Viva Vida programme (see section 11c), participants identified with a communication pattern that might indicate that our WhatsApp number was blocked (consecutive WhatsApp messages not delivered to participants) will be removed from our broadcast list to abide by WhatsApp policy and to keep our system functional. No new intervention messages will be sent, but participants will be contacted for the follow-up assessment, unless they withdraw their consent for follow up.

Strategies to improve adherence to interventions (11c)

During the first two weeks participants receive the intervention, the research team will actively identify those who might have an issue receiving or opening the messages, based on the status of the WhatsApp messages, and contact them by phone. After this period, no other strategies will be adopted. Participants who have technical difficulties using the application will be able to contact a dedicated phone number to try to solve the issue with support from the research team throughout the whole period of the trial.

Relevant concomitant care permitted or prohibited during the trial (11d)

Neither ongoing nor new pharmacological or non-pharmacological treatments, nor health appointments, will be prohibited during the trial.

Outcomes (12)

Primary outcome

We will assess the proportion of participants recovered from depression with the PHQ-9 (15, 16) (PHQ-9 score<10), three months after receiving the first message (intervention group) or the single message (control group).

Secondary outcomes

The proportion recovered at five months (PHQ-9<10) will assess the maintenance of any earlier clinical gains. The continuous score of the PHQ-9 will also be considered at three and five months, as well as, anxiety symptomatology assessed with the Generalized Anxiety

Disorder-7 (GAD-7) (17) and loneliness assessed with the 3-item University of California, Los Angeles (UCLA) loneliness scale (3-item UCLA) (18).

Cost-effectiveness outcomes

Assessment of quality of life with the European Quality of Life five-dimensional questionnaire, five-level version (EQ-5D-5L) (19), and capability wellbeing with the ICEpop CAPability measure for Older people (ICECAP-O) (20) at four and eight months.

Implementation outcomes

We will use qualitative methods to assess: (a) the acceptability, appropriateness, and feasibility of the intervention by participants; (b) whether the intervention was received by participants as intended (fidelity); (c) the participants' contextual barriers and enablers to the implementation of the intervention; and (d) the relationship between implementation outcomes and clinical effectiveness.

Participant timeline (13)

Participants will be randomised after granting consent to participate in the trial and will be included in a list to start receiving the messages (both control and intervention arms) no more than 10 days after consenting to participate. Each list will include participants recruited during the previous week who will receive the single message (control group) or the first message (intervention group) on the following Monday. Participants allocated to the intervention arm will receive messages every Monday, Wednesday, Friday and Saturday twice a day during six weeks. The follow-up assessment will occur at three (weeks 12 to 16) and five months (weeks 20 to 24) after receiving the first message. Figure 1 shows the complete timeline of the study.

Sample size (14)

A sample size of 330-374 individuals will detect a 15 percentage point difference (25% versus 40%) in recovery between the control and intervention groups after three months, with 80-85% power and two-sided 5% significance level. Such a difference in recovery rate is considered clinically meaningful (21-25). We anticipate 25% attrition, which is readily achievable according to the experience we had collecting follow-up data by phone in our previous RCT in Guarulhos, yielding a corrected total sample size of 440-500.

All of the following estimates are based on the PROACTIVE RCT (5) and our pilot study in São Paulo (26). We expect that on average 10% of the individuals registered with each UBS will be aged 60 years or older. On average, there are approximately 1,600 individuals in the eligible age range registered with each UBS. Based on the PROACTIVE data, we would be able to reach 440 individuals by phone and 60% of them (250 individuals) would have a mobile phone and would use WhatsApp application. With a proportion of depressive symptomatology (PHQ-9≥10) of around 20% (26, 27), there would be 50 individuals potentially eligible in each UBS, though this is likely to reduce to 35 once other entry criteria are applied.

We will work with at least 24 UBSs, and we plan to complete recruitment in six months. We will need to screen approximately 25,000 individuals to achieve a sample of 500 participants. Of these 25,000 individuals, we expect that we will be able to contact 6,800. Among the individuals we can contact, 3,800 will use WhatsApp. We expect that at least 20% will have depressive symptomatology (n=760), and 530 will consent to participate in the study – that is, at the upper end of the above range from our sample size calculation.

Recruitment (15)

A list with names and contact details of all individuals aged 59 years or over and registered within the 24 UBSs will be provided by the Health Secretariat of Guarulhos. We will exclude duplicated names and PROACTIVE participants from the list. All individuals in the list will receive a random ID. These IDs will be ordered before entering the study system and the recruitment will follow this order. We will first approach individuals by sending WhatsApp messages to those who have at least one mobile phone number. The message will give a brief introduction to the study and will inform individuals that a research assistant will contact them soon. Initially, only those whose WhatsApp message was successfully delivered will be contacted.

The recruitment comprises three parts: (a) screening; (b) baseline; and (c) invitation to participate in the study. Screening and baseline assessment will be carried out consecutively where individuals will be invited to participate in the study during the same phone call, whenever it is possible. The following are details of each stage of the recruitment process:

a. Screening: assessment of inclusion criteria. Before starting the screening, we will inform the participant about the interview and seek consent. Then, we will confirm the age and the use of WhatsApp. Individuals will be screened with the first two questions of the PHQ-9 (PHQ-2). If they present at least one core symptom of depression (depression mood and

anhedonia, i.e. PHQ-2≥1) (28), the evaluation of depressive symptomatology will be completed with the remaining seven questions (11).

b. Baseline: Older adults who use WhatsApp, with PHQ-9≥10 (cut-off for depressive symptomatology) (11) and who do not present acute suicidal risk (as assessed by the 9th item of the PHQ-9 and the Immediate Suicide Risk Protocol) or any other exclusion criteria will complete a full baseline assessment.

c. Invitation to participate in the clinical trial: after completing the baseline assessment, the research assistant will provide information about the study (type of interventions and follow-up assessments) for all eligible individuals and invite them to participate in the study. Additionally, the WhatsApp number will be 'revalidated' by sending and confirming the receipt of a 'Welcome' message.

Assignment of interventions: allocation

Sequence generation (16a)

Participants will be randomly allocated in a 1:1 ratio to either intervention or control arms. Stratification will be based on the PHQ-9 groups (scores of 10-14, 15-19 and 20+), gender (male and female) and age groups (60-69, 70-79 and 80+ years). Random permuted blocks with random block sizes will be used to generate the randomisation sequence with the support of Microsoft Excel.

Concealment mechanism (16b)

The randomisation sequence will be concealed in the "randomization module" of the Research Electronic Data Capture (REDCap).

Implementation (16c)

The allocation sequence will be generated by members of the research team (CAN and TJP) not directly involved in recruiting individual participants, who will be enrolled by an independent research assistant. Data collected will be reviewed by the research coordinator after the participant's consent to participate in the study and the study arm will be allocated with the support of REDCap. The intervention team will receive the list of randomised participants and will send the allocated messages.

Assignment of interventions: Blinding

Who will be blinded (17a)

Given the difference between the number and contents of the messages received by the intervention and control groups, blinding of participants will not be feasible. A list with the participants' allocation group will be sent every week to the researchers responsible for sending the messages. Research assistants will be completely blind to the group allocation during the follow-up assessments and, whenever possible, the research assistant will not carry out more than one assessment (recruitment and follow-ups) with the same participant. The research teams that coordinate the recruitment and intervention will work separately. The assessments and the interventions will be delivered remotely and individually in order to minimise the risk of contamination.

Procedure for unblinding if needed (17b)

No procedure for unblinding is planned for this trial.

Data collection and management

Plans for assessment and collection of outcomes (18a)

Recruitment and the 3- and 5-month follow-up interviews will be carried out by independent research assistants through phone calls. The research assistants will be trained to conduct the assessments and to use the data collection software (REDCap). Quality control of a sample of recorded assessments will be conducted by an independent research assistant. Qualitative assessments will be carried out while participants are receiving the intervention and after the first follow-up assessment.

Instruments

Depressive symptomatology will be assessed using the PHQ-9 (11). This instrument was chosen as it is widely used and there is evidence of validity in Brazil (29, 30). Anxiety symptomatology will be initially screened with the first two questions of the GAD-7 (17) and if GAD-2≥1 (31), the remaining five questions will be assessed. The 3-item UCLA will be used to assess loneliness (18). Quality of Life will be assessed with the EQ-5D-5L (19) and the ICECAP-O (20). Level of depressive symptomatology was associated with these two measures in our previous study (32). Physical and chronic conditions, depression treatment, sociodemographic profile (level of education, personal and household income, marital status, race and living arrangement), alcohol and tobacco use, and use of WhatsApp will also be asked during the recruitment assessment.

During both follow-up assessments, questionnaires completed at baseline will be repeated including the PHQ-9, GAD-7, 3-item UCLA, EQ-5D-5L and ICECAP-O. Participants will also be asked about admissions to hospital and consultations related to mental healthcare and any ongoing treatment for depression. Additionally, questions about the experience receiving the messages will be included for the intervention arm. For every participant, we will request the electronic health record system data on psychotropic medications and consultations with doctors and nurses during the period they were participating in the trial.

Plans to promote participant retention and complete follow-up (18b)

For each follow-up, we will adopt a 4-week window in order to maximise our chances to complete the follow-up assessments. Whenever we have issues with contacting the participant by phone call, we will first send him/her an audio and/or a text message by WhatsApp with a reminder about the assessment. If the participant does not reply to these messages, the UBS manager or an appointed staff member will be contacted to help us reach him/her. If we are unable to find the participant by phone within the first three weeks of the follow-up window, a research assistant will attempt to visit the participant at home to complete a face-to-face assessment.

Data management (19)

All assessment data will be collected and managed using REDCap (33, 34) hosted at the Hospital das Clínicas da Faculdade de Medicina da Universidade de Sao Paulo. REDCap is a secure, web-based software platform designed to support data capture for research studies, providing: 1) an intuitive interface for validated data capture; 2) audit trails for tracking data manipulation and export procedures; 3) automated export procedures for seamless data downloads to common statistical packages; and 4) procedures for data integration and interoperability with external sources.

Statistical methods

Statistical methods for primary and secondary outcomes (20a)

The analysis for all outcome measures will follow the intention-to-treat principle and adhere to Consolidated Standards of Reporting Trials guidelines for randomised trials (35). To understand if there are any imbalances in risk factors for depression, descriptive statistics will be used to compare differences in baseline characteristics between treatment arms.

All outcomes will be evaluated using regression models that adjust for stratification, the baseline assessment of the corresponding outcome measure. For secondary analyses only, any baseline variables that are not balanced between treatment arms will be accounted for in these models. Logistic regression models will be used to evaluate for the primary outcome (recovery from depression at four months) and the secondary outcome (recovery from depression at eight months). Odds ratios with 95% confidence intervals will be used to estimate recovery from depression at four and eight months. Other secondary outcomes including PHQ-9, GAD-7, 3-item UCLA, EQ-5D-5L and ICECAP-O scores will be assessed using linear regression models. Coefficients and associated 95% confidence intervals for these outcomes represent differences in means between the intervention and control arms. Sensitivity analyses will be conducted to investigate any potential clustering at the USB level.

Economic analysis

Two economic analyses will be conducted. 1) Incremental cost-effectiveness between the two arms of the trial will be estimated using: a) the primary clinical outcome measure (cost per patient recovered); and b) Quality Adjusted Life Years (QALYs) calculated using the EQ-5D-5L (36, 37). These results will be presented as incremental cost-effectiveness ratios and cost-effectiveness acceptability curves. These show the probability of the intervention being cost-effective at a range of 'willingness-to-pay' threshold levels. 2) The net monetary benefit statistic (NMB), using the difference in costs and the difference in QALYs between the two arms, will be calculated at the WHO recommended threshold for LMICs.

We will conduct the cost-effectiveness analysis from the health system perspective to compare the costs and effects of the 12-week digital psychosocial programme against a single message. The EQ-5D-5L (36, 38, 39) responses will be converted into utility scores using the population tariff most appropriate for the Brazilian population available at the time of the analysis. Utility scores will be used to estimate QALYs, adjusting for baseline values. We will use national, where available, or local unit costs to value resource use.

Process evaluation

Two assessments of implementation outcomes will be conducted by phone using qualitative interviews. These interviews will provide insight into the reasons, motivations, modes, and contexts of participants from intervention arm that may affect their clinical and

implementation outcomes, as well as the detailed process and content perspectives of how the intervention was received by participants. Approximately 24 participants who received the intervention will be purposively selected for the first assessment, conducted individually, 15 to 30 days after the 3-month follow-up assessment. We will ensure adequate representation from the following subgroups: gender (women and men), age (60-69 years old and 70 years or over), depression status at follow-up 1 (recovered and not recovered from depression). For the second assessment, we will recruit an extra group of 15 individuals using the same inclusion and exclusion criteria of the trial. They will not be included in the main trial analyses so as to eliminate any potential influence these interviews may have on depression outcomes. They will be interviewed weekly by the same research assistant to assess key implementation outcomes throughout the course of the 6-week Viva Vida Programme as well as exploring participants' contextual and behavioural barriers/enablers to the implementation of the intervention. Informed consent to participate and the qualitative assessments will be audio recorded if the participant allows. Interviews will last around 30 minutes.

Qualitative analysis

All interviews will be audio-recorded, transcribed and analysis will be conducted using Atlas.TI software. The qualitative assessment applied between follow-up assessments will be thematically analysed deductively using pre-established categories (acceptability, appropriateness, fidelity, and feasibility), as well as inductively for other relevant categories emerging from the analysis. In-depth exploration of participants' experiences will enable us the generation of hypothetical propositions about the relationship between clinical outcomes (considering the PHQ-9 score assessed at the beginning of the study and in the follow-up assessment) and implementation outcomes. Analysis of narratives and images collected during the intervention will also be thematically analysed to understand the user journey (including behavioural activation, contextual barriers, facilitators. etc.) as well as how participants' experiences changed over the six weeks of receiving the intervention.

Methods for additional analyses (e.g. subgroup analyses) (20b)

A Complier Average Case Effect (CACE) analysis will be used to determine the effect of number of messages listened to on recovery from depression at both four and eight months. A threshold value (listening to most of the messages received) will be used for the CACE

analysis with PHQ-9 scores at both time points. We will use this threshold as it was hypothesised to be the minimum number of sessions needed to have a therapeutic effect. A sensitivity analysis using the thresholds of listening to at least half of the messages or all messages will be conducted, and we will also consider using the number of messages opened to as a continuous variable unless the relevant relationship is far from linear.

Additional exploratory subgroup analyses will be used to estimate whether the following pre-specified characteristics of the participants modified the effect of the intervention: baseline PHQ-9; gender; age; education level; presence of co-morbid physical illnesses. To test for modification, an interaction term will be introduced between the pre-specified variable and the treatment arm. Results of these analyses will need to be interpreted with caution due to the limited power to detect such interactions as well as the paucity of evidence on the theoretical basis for these hypotheses.

Methods in analysis to handle protocol non-adherence and any statistical methods to handle missing data (20c)

Patterns and the proportion of missing data for the primary and secondary outcomes will be investigated. If there are any marked differences in missing data between treatment arms, or if greater than 10% of the data are missing, multiple imputation using chained equations (MICE models) under the assumption that data is missing at random (MAR) will be used. Data will be imputed separately for the different treatment arms and MICE models will include any variables that predict missingness. Sensitivity analyses testing for modest departures against the MAR assumption will be conducted using the Selection Model Approach.

Composition of the Data Monitoring Committee, its role and reporting structure (21a)

Data Monitoring Committee (DMC)

Under the guidance of the TSC, a Data Monitoring Committee (DMC) will be formed comprising an independent Chair, an independent statistician, one other independent member, and at least one of the trial statisticians.

Interim analyses (21b)

No interim analyses are planned for this trial.

Adverse event reporting and harms (22)

The risk associated with the trial and the digital intervention are considered minimal. Participants will receive any pharmacological and non-pharmacological treatment they were prescribed before or during the study. Also, participants of both arms will receive messages advising them to seek healthcare if the symptoms are not improving or if they are worsening. Identification of acute suicide risk at recruitment or follow-up assessments will activate a standardised protocol, and a family member and the UBS manager will be contacted by the research team. This protocol was successfully applied in the PROACTIVE study (5, 26). Individuals identified with an acute suicide risk during the recruitment assessment will not be eligible to participate in the study.

Frequency and plans for auditing trial conduct (23)

Characteristics used for stratification of randomisation will be monitored in order to ensure that the allocation sequence and REDCap “randomization module” are working as expected, and the two study arms are reasonably well balanced in each stratum. The TSC will be presented with a report of the trial conduct.

Ethics approval and consent to participate (24)

Ethical approval was sought from the Ethics Committee of the Hospital das Clínicas da Faculdade de Medicina da Universidade de Sao Paulo – HCFMUSP (CAPPesq, ref: 4.097.596, first approved on 10th March 2021). This study was also authorised by the Secretaria da Saúde do Município de Guarulhos.

Plans for communicating important protocol amendments to relevant parties (25)

Modifications on the study protocol will be discussed between the research team, TSC and DMC and submitted to approval of the Ethics Committee. Whenever relevant, Guarulhos health system managers and coordinators will be consulted before any decision.

Who will take informed consent? (26a)

Verbal informed consent will be sought by the research assistant before screening and qualitative assessments and when inviting the eligible individuals to participate in the study. All assessments will be conducted by phone. Verbal consent and assessments will be recorded whenever authorised by the individual.

Additional consent provisions for collection and use of participant data and biological specimens (26b)

No additional consent for collection and use of participant data and biological specimens will be collected.

Confidentiality (27)

This study will be compliant to the National Commission of Ethics in Research (Comissão Nacional de Ética em Pesquisa), Resolution 466/2012 security policies. One member of the team will extract the individual's information from the list provided by the Health Secretariat of Guarulhos, and then they will receive a random ID number. The same ID number will be used when entering data into the REDCap platform and the system developed to deliver the messages. The access to both systems is secured by password and limited according to the members of the research team's assigned roles and permissions. Audio files of recruitment, follow-up and qualitative assessments will be renamed using the corresponding ID number and initials and stored on a password-protected platform. Only the recruitment coordination team will have access to these audio files. No paper records will be kept.

Declaration of interests (28)

The authors have no conflict of interest to declare.

Access to data (29)

Study documentation and trial data will be available on request according to data sharing conditions.

Ancillary and post-trial care (30)

No post-trial care is planned for this study.

Dissemination policy (31a)

Publications on internationally competitive journals and presentation at relevant conferences are expected from this trial. Meetings with the main stakeholders will be organised at the end of the trial to present the main results and discuss further steps and potential future collaboration.

Authors' contributions (31b)

Plans to give access to the full protocol, participant level-data and statistical code (31c)

Participant level-data and statistical code will be available for external use 24 months after publishing the paper with effectiveness results. A research proposal with defined aims and a statistical analysis plan should accompany any request and all requests will be evaluated by the joint principal investigators before granting access to the data.

Consent for publication (32)

Participants will be advised that non-identifiable data will be used for publications before granting consent in both assessments.

Plans for collection, laboratory evaluation and storage of biological specimens for genetic or molecular analysis in this trial/future use (33)

No biological specimens will be collected during this study.

References

1. Rathod S, Pinninti N, Irfan M, Gorczynski P, Rathod P, Gega L, et al. Mental Health Service Provision in Low- and Middle-Income Countries. Health Serv Insights. 2017;10:1178632917694350.
2. World Health Organization. Depression and Other Common Mental Disorders. Global Health Estimates.2017. Available from: http://apps.who.int/iris/bitstream/handle/10665/254610/WHO-MSD-MER-2017.2-eng.pdf. Accessed 01 March 2022.
3. World Health Organization. Mental health of older adults. 2017. https://www.who.int/news-room/fact-sheets/detail/mental-health-of-older-adults. Accessed 01 March 2022.
4. World Federation for Mental Health. Depression: a global crisis. World Mental Health Day, October 10 20122012. Available from: https://www.researchgate.net/publication/285075782_Depression_A_global_public_health_concern. Accessed 01 March 2022.
5. Scazufca M, Nakamura CA, Peters TJ, Henrique MG, Seabra A, La Rotta EG, et al. A collaborative care psychosocial intervention to improve late life depression in socioeconomically deprived areas of Guarulhos, Brazil: the PROACTIVE cluster randomised controlled trial protocol. Trials. 2020;21(1):914.
6. Van de Ven P, Araya R, P de Paula Couto MC, Henrique MG, Meere D, Vilela Mendes A, et al. Investigating Software Requirements for Systems Supporting Task-Shifted Interventions: Usability Study. J Med Internet Res. 2019;21(11):e11346.
7. Firth J, Torous J, Nicholas J, Carney R, Pratap A, Rosenbaum S, et al. The efficacy of smartphone-based mental health interventions for depressive symptoms: a meta-analysis of randomized controlled trials. World Psychiatry. 2017;16(3):287-98.
8. Fu Z, Burger H, Arjadi R, Bockting CLH. Effectiveness of digital psychological interventions for mental health problems in low-income and middle-income countries: a systematic review and meta-analysis. Lancet Psychiatry. 2020;7(10):851-64.
9. Araya R, Menezes PR, Claro HG, Brandt LR, Daley KL, Quayle J, et al. Effect of a Digital Intervention on Depressive Symptoms in Patients With Comorbid Hypertension or Diabetes in Brazil and Peru: Two Randomized Clinical Trials. JAMA. 2021;325(18):1852-62.
10. Riadi I, Kervin L, Teo K, Churchill R, Cosco TD. Digital Interventions for Depression and Anxiety in Older Adults: Protocol for a Systematic Review. JMIR Res Protoc. 2020;9(12):e22738.
11. Kroenke K, Spitzer RL, Williams JB. The PHQ-9: validity of a brief depression severity measure. J Gen Intern Med. 2001;16(9):606-13.
12. Carswell K, Harper-Shehadeh M, Watts S, Van't Hof E, Abi Ramia J, Heim E, et al. Step-by-Step: a new WHO digital mental health intervention for depression. Mhealth. 2018;4:34.
13. Mehrotra S, Tripathi R. Recent developments in the use of smartphone interventions for mental health. Curr Opin Psychiatry. 2018;31(5):379-88.
14. Chen YR, Schulz PJ. The Effect of Information Communication Technology Interventions on Reducing Social Isolation in the Elderly: A Systematic Review. J Med Internet Res. 2016;18(1):e18.
15. Lowe B, Unutzer J, Callahan CM, Perkins AJ, Kroenke K. Monitoring depression treatment outcomes with the patient health questionnaire-9. MedCare. 2004;42(12):1194-201.
16. Kroenke K, Spitzer RL, Williams JB. The PHQ-9: validity of a brief depression severity measure. JGenInternMed. 2001;16(9):606-13.
17. Spitzer RL, Kroenke K, Williams JB, Löwe B. A brief measure for assessing generalized anxiety disorder: the GAD-7. Arch Intern Med. 2006;166(10):1092-7.
18. Hughes ME, Waite LJ, Hawkley LC, Cacioppo JT. A Short Scale for Measuring Loneliness in Large Surveys: Results From Two Population-Based Studies. Res Aging. 2004;26(6):655-72.
19. Devlin NJ, Krabbe PF. The development of new research methods for the valuation of EQ-5D-5L. Eur J Health Econ. 2013;14 Suppl 1:S1-3.
20. Grewal I, Lewis J, Flynn T, Brown J, Bond J, Coast J. Developing attributes for a generic quality of life measure for older people: preferences or capabilities? Soc Sci Med. 2006;62(8):1891-901.

21. Ciechanowski P, Wagner E, Schmaling K, Schwartz S, Williams B, Diehr P, et al. Community-integrated home-based depression treatment in older adults: a randomized controlled trial. JAMA. 2004;291(13):1569-77.
22. Araya R, Rojas G, Fritsch R, Gaete J, Rojas M, Simon G, et al. Treating depression in primary care in low-income women in Santiago, Chile: a randomised controlled trial. Lancet. 2003;361(9362):995-1000.
23. Unutzer J, Katon W, Callahan CM, Williams JW, Jr., Hunkeler E, Harpole L, et al. Collaborative care management of late-life depression in the primary care setting: a randomized controlled trial. JAMA. 2002;288(22):2836-45.
24. Cramer H, Salisbury C, Conrad J, Eldred J, Araya R. Group cognitive behavioural therapy for women with depression: pilot and feasibility study for a randomised controlled trial using mixed methods. BMC Psychiatry. 2011;13.
25. Richards DA, Hill JJ, Gask L, Lovell K, Chew-Graham C, Bower P, et al. Clinical effectiveness of collaborative care for depression in UK primary care (CADET): cluster randomised controlled trial. BMJ. 2013;347:f4913.
26. Scazufca M, de Paula Couto MCP, Henrique MG, Mendes AV, Matijasevich A, Pereda PC, et al. Pilot study of a two-arm non-randomized controlled cluster trial of a psychosocial intervention to improve late life depression in socioeconomically deprived areas of São Paulo, Brazil (PROACTIVE): feasibility study of a psychosocial intervention for late life depression in São Paulo. BMC Public Health. 2019;19(1):1152.
27. Nakamura CA, Scazufca M, Peters TJ, Fajersztajn L, Van de Ven P, Hollingworth W, et al. Depressive and subthreshold depressive symptomatology among older adults in a socioeconomically deprived area in Brazil. Int J Geriatr Psychiatry. 2022;37(2).
28. Kroenke K, Spitzer RL, Williams JB. The Patient Health Questionnaire-2: validity of a two-item depression screener. Med Care. 2003;41(11):1284-92.
29. Santos IS, Tavares BF, Munhoz TN, Almeida LS, Silva NT, Tams BD, et al. [Sensitivity and specificity of the Patient Health Questionnaire-9 (PHQ-9) among adults from the general population]. Cad Saude Publica. 2013;29(8):1533-43.
30. Moreno-Agostino D, Chua KC, Peters TJ, Scazufca M, Araya R. Psychometric properties of the PHQ-9 measure of depression among Brazilian older adults. Aging Ment Health. 2021:1-6.
31. Kroenke K, Spitzer RL, Williams JB, Monahan PO, Löwe B. Anxiety disorders in primary care: prevalence, impairment, comorbidity, and detection. Ann Intern Med. 2007;146(5):317-25.
32. Nakamura CA, Mitchell PM, Peters TJ, Moreno-Agostino D, Araya R, Scazufca M, et al. A Validation Study of the EQ-5D-5L and ICEpop Capability Measure for Older People Among Older Individuals With Depressive Symptoms in Brazil. Value in Health Regional Issues. 2022;30:91-9.
33. Harris PA, Taylor R, Thielke R, Payne J, Gonzalez N, Conde JG. Research electronic data capture (REDCap)--a metadata-driven methodology and workflow process for providing translational research informatics support. J Biomed Inform. 2009;42(2):377-81.
34. Harris PA, Taylor R, Minor BL, Elliott V, Fernandez M, O'Neal L, et al. The REDCap consortium: Building an international community of software platform partners. J Biomed Inform. 2019;95:103208.
35. Schulz KF, Altman DG, Moher D, Group C. CONSORT 2010 Statement: updated guidelines for reporting parallel group randomised trials. Trials. 2010;11:32.
36. Williams A, Kind P. The present state of play about QALYs. In: Hopkins A, editor. Measures of the quality of life and the uses to which such measures may be put. London: RCP Publications; 1992.
37. Devlin NJ, Krabbe PF. The development of new research methods for the valuation of EQ-5D-5L. The European journal of health economics : HEPAC : health economics in prevention and care. 2013;14:S1-S3.
38. Sapin C, Fantino B, Nowicki ML, Kind P. Usefulness of EQ-5D in Assessing Health Status in Primary Care Patients with Major Depressive Disorder. Health and Quality of Life Outcomes. 2004;2:20.
39. Viegas Andrade M, Noronha K, Kind P, Maia AC, Miranda de Menezes R, De Barros Reis C, et al. Societal Preferences for EQ-5D Health States from a Brazilian Population Survey. Value in Health Regional Issues. 2013;2:405-12.

Summary of amendments

Version*	Date	Changes
1	08/02/2021	Initial protocol
2	17/07/2021	Reduction of intervention duration from ‘12 weeks’ to ‘six weeks’, and changes to follow-ups visits from ‘four and eight months’ to ‘three and five months’ after the pilot study. Description of the developed system to deliver the intervention.
3	07/12/2021	Addition of qualitative interviews after the first follow-up to collect data for process evaluation
4	09/02/2022	Correction of consent form requested by the Ethics Committee
5	16/03/2022	Final protocol

* Based on Ethics Committee documentation (Plataforma Brasil – CAAE 43158321.5.0000.0068)
