# Supplementary material for: Self-help mobile messaging intervention for depression among older adults in resource-limited settings: a randomized controlled trial
Source: Nat Med. 2024 Mar 14;30(4):1127–33. doi: 10.1038/s41591-024-02864-4 (PMC11031393; doi:10.1038/s41591-024-02864-4)
Supplement: Supplementary file 2 — Reporting Summary [file 41591_2024_2864_MOESM2_ESM.pdf]

nature portfolio

Corresponding author(s): Marcia Scazufca

Last updated by author(s): Jan 30, 2024

Reporting Summary

Nature Portfolio wishes to improve the reproducibility of the work that we publish. This form provides structure for consistency and transparency in reporting. For further information on Nature Portfolio policies, see our Editorial Policies and the Editorial Policy Checklist.

Statistics

For all statistical analyses, confirm that the following items are present in the figure legend, table legend, main text, or Methods section.

n/a	Confirmed	
☐	☒	The exact sample size (*n*) for each experimental group/condition, given as a discrete number and unit of measurement
☒	☐	A statement on whether measurements were taken from distinct samples or whether the same sample was measured repeatedly
☐	☒	The statistical test(s) used AND whether they are one- or two-sided<br>*Only common tests should be described solely by name; describe more complex techniques in the Methods section.*
☐	☒	A description of all covariates tested
☐	☒	A description of any assumptions or corrections, such as tests of normality and adjustment for multiple comparisons
☐	☒	A full description of the statistical parameters including central tendency (e.g. means) or other basic estimates (e.g. regression coefficient) AND variation (e.g. standard deviation) or associated estimates of uncertainty (e.g. confidence intervals)
☐	☒	For null hypothesis testing, the test statistic (e.g. *F*, *t*, *r*) with confidence intervals, effect sizes, degrees of freedom and *P* value noted<br>*Give P values as exact values whenever suitable.*
☒	☐	For Bayesian analysis, information on the choice of priors and Markov chain Monte Carlo settings
☒	☐	For hierarchical and complex designs, identification of the appropriate level for tests and full reporting of outcomes
☐	☒	Estimates of effect sizes (e.g. Cohen's *d*, Pearson's *r*), indicating how they were calculated

Our web collection on statistics for biologists contains articles on many of the points above.

Software and code

Policy information about availability of computer code

Data collection	REDCap 13.7.9, WhatsApp Business API 22.4.3 to 22.7.2
Data analysis	Stata 17

For manuscripts utilizing custom algorithms or software that are central to the research but not yet described in published literature, software must be made available to editors and reviewers. We strongly encourage code deposition in a community repository (e.g. GitHub). See the Nature Portfolio guidelines for submitting code & software for further information.

Data

Policy information about availability of data

All manuscripts must include a data availability statement. This statement should provide the following information, where applicable:

- Accession codes, unique identifiers, or web links for publicly available datasets
- A description of any restrictions on data availability
- For clinical datasets or third party data, please ensure that the statement adheres to our policy

De-identified individual participant data and data dictionary will be made available 24 months after publication. Proposals with specific aims and an analysis plan should be directed to the corresponding author.

Research involving human participants, their data, or biological material

Policy information about studies with human participants or human data. See also policy information about sex, gender (identity/presentation), and sexual orientation and race, ethnicity and racism.

Reporting on sex and gender	Gender was self-reported.<br>Subgroup analyses, including for gender, are also reported in the study.
Reporting on race, ethnicity, or other socially relevant groupings	This study does not report any information on race or ethnicity.
Population characteristics	The majority of participants were women (75%), aged 60-69 year-old (83%), with less than eight years of education (74%) and were earning minimum wage (64%). Severity of depressive symptomatology was considered moderate (PHQ-9 scores between 10 and 14) for 40% of the participants.
Recruitment	Participants were recruited by phone following a list of individuals registered with the participating primary care clinics. Individuals with an active WhatsApp number were approached by telephone to be screened for depressive symptomatology using the Patient Health Questionnaire (PHQ). Those with PHQ-2 scores≥1 were administered the full PHQ-9 and if they scored ≥10 as well as met our other eligibility criteria they were invited to participate in the trial. The list of individuals were provided by the Guarulhos Health Secretariat and we pre-screened 95% of all registered individuals. Around 72% of those with a valid WhatsApp number could not be included as they did not answer either of the two calls made by the research assistants (these calls were on different days but logistical constraints precluded more than two such attempts). Possible reasons for failure to answer the call include that the telephone was turned off, the call was not heard, or the individual did not answer calls from unknown numbers. There is therefore potential for an element of self-selection in this part of the recruitment process although this would not have impacted appreciably on the internal validity of the cluster randomised trial.
Ethics oversight	Ethics Committee of the Hospital das Clínicas da Faculdade de Medicina da Universidade de Sao Paulo – HCFMUSP

Note that full information on the approval of the study protocol must also be provided in the manuscript.

Field-specific reporting

Please select the one below that is the best fit for your research. If you are not sure, read the appropriate sections before making your selection.

☐ Life sciences ☒ Behavioural & social sciences ☐ Ecological, evolutionary & environmental sciences

For a reference copy of the document with all sections, see nature.com/documents/nr-reporting-summary-flat.pdf

Behavioural & social sciences study design

All studies must disclose on these points even when the disclosure is negative.

Study description	PRODIGITAL-D was a two-arm individually-randomized controlled trial with 1:1 allocation. This study reports the effectiveness outcomes of the trial.
Research sample	Older adults (60+ years) registered with a primary care clinic in Guarulhos, Brazil, and with a valid phone and WhatsApp number. The majority of participants were women (75%) and aged 60-69 year-old (83%). The majority had less than eight years of education (74%) and were earning minimum wage (64%). The sample is representative of the older population with depressive symptoms in Brazil. The Viva Vida program was developed to reach older adults registered with primary care clinics, commonly located in socioeconomically deprived areas of the country.
Sampling strategy	Random sampling was employed to recruit participants. Sample size was calculated considering that 440-500 randomized individuals would yield 80-85% power to detect a 15-percentage point difference in depression recovery rates between the control and intervention arms at three months (25% versus 40%) using a two-sided 5% alpha, and assuming a 25% attrition.
Data collection	The following instruments were used during the baseline and follow-up assessments: 9-item Patient Health Questionnaire (PHQ-9), Generalized Anxiety Disorder-7 (GAD-7), 3-item University of California, Los Angeles (UCLA) loneliness scale (3-item UCLA), European Quality of Life five-dimensional questionnaire, five-level version (EQ-5D-5L); ICEpop CAPability measure for Older people (ICECAP-O). All data were collected by phone calls and managed using REDCap. Research assistants involved in recruitment and follow-up data collection were masked to trial allocation. Two different groups of research assistants were responsible for either the first or the second follow-up assessment.
Timing	Data collection took place between 8 September 2021 and 26 September 2022.
Data exclusions	No data were excluded from the analyses.
Non-participation	No participants dropped out/declined participating after randomisation.

Randomization	Participants were stratified by age group (60-69 years, 70-79, 80+ years), gender (male, female) and depression severity (PHQ-9 categories: 10-14, 15-19, 20+). The allocation sequence was generated using random-permuted blocks with random block sizes by research team members not involved in data collection. The 'randomization module' of the Research Electronic Data Capture (REDCap) was used to conceal the allocation sequence and randomize individuals.

Reporting for specific materials, systems and methods

We require information from authors about some types of materials, experimental systems and methods used in many studies. Here, indicate whether each material, system or method listed is relevant to your study. If you are not sure if a list item applies to your research, read the appropriate section before selecting a response.

Materials & experimental systems

n/a	Involved in the study
☒	☐ Antibodies
☒	☐ Eukaryotic cell lines
☒	☐ Palaeontology and archaeology
☒	☐ Animals and other organisms
☐	☒ Clinical data
☒	☐ Dual use research of concern
☒	☐ Plants

Methods

n/a	Involved in the study
☒	☐ ChIP-seq
☒	☐ Flow cytometry
☒	☐ MRI-based neuroimaging

Clinical data

Policy information about clinical studies

All manuscripts should comply with the ICMJE guidelines for publication of clinical research and a completed CONSORT checklist must be included with all submissions.

Clinical trial registration	Brazilian Registry of Clinical Trials (ReBEC), RBR-4c94dtn.
Study protocol	Full trial protocol is part of the supplemental material.
Data collection	Participants were recruited by phone from a randomly ordered list of 24 primary care clinics, named Unidades Básicas de Saúde (UBSs), in Guarulhos, Brazil. The city is part of the metropolitan region of Sao Paulo with a population of around 1.4 million. A total of 603 participants were recruited between September 2021 and April 2022, and followed up for five months.
Outcomes	Recovery from depression (PHQ-9 score<10) at three months was the primary outcome. Secondary outcomes included recovery from depression at five months, and reduction in PHQ-9 scores by at least 50% between baseline and follow-up visits at three and five months. We also evaluated effects on the continuous scores of: anxiety symptomatology with the Generalized Anxiety Disorder-7 (GAD-7); loneliness with the 3-item University of California, Los Angeles (UCLA) loneliness scale (3-item UCLA); quality of life with the European Quality of Life five-dimensional questionnaire, five-level version (EQ-5D-5L); and capability wellbeing with the ICEpop CAPability measure for Older people (ICECAP-O).
